# Synthesis, characterization and photocatalytic evaluation of ternary metal–selenide-chitosan microspheres for the degradation of Congo red dye

**DOI:** 10.1039/d6ra04309g

**Published:** 2026-07-31

**Authors:** Nawaz Khan, Waqar Ahmad, Muhammad Khan, Mahnoor Amjad, Adnan Khan, Sabir Khan, Nisar Ali, Nauman Ali, Jiaojiao Zheng

**Affiliations:** a Institute of Chemical Sciences, University of Peshawar Khyber Pakhtunkhwa 25120 Pakistan; b Energy & Bioproducts Research Institute (EBRI), College of Engineering and Physical Sciences (EPS), Aston University Aston Triangle Birmingham B4 7ET UK j.zheng2@aston.ac.uk; c Center of Chemical, Pharmaceutical and Food Sciences, Federal University of Pelotas Pelotas 96010-900 RS Brazil; d Materials Science and Engineering (PPGCEM), Technological Development Center, Federal, University of Pelotas (UFPel) Pelotas 96010-610 RS Brazil; e Department of Basic and Applied Sciences, College of Applied and Health Sciences, A'Sharqiyah University P.O. Box 42 Ibra P.O. 400 Sultanate of Oman

## Abstract

In this study, ternary nickel–chromium selenide (NiCrSe)-chitosan microspheres were synthesized *via* a co-precipitation approach and evaluated as an efficient visible-light-driven photocatalyst for the degradation of Congo red (CR) dye. The structural, morphological and compositional properties of the composite were confirmed by FTIR, XRD, SEM-EDX and zeta-potential analyses, which revealed well-defined nanostructured features. The catalyst exhibited an optical band gap of 2.2 eV, indicating strong absorption in the visible region and good potential for solar-driven photocatalysis. Under optimized conditions (0.05 g catalyst dosage, 10 ppm dye concentration, pH 6 and 60 min solar irradiation), a maximum degradation efficiency of approximately 97% was achieved. The process was optimized using Response Surface Methodology (RSM), which revealed significant individual and interactive effects of the operating parameters, with catalyst dosage being the most influential. The quadratic model showed excellent statistical reliability (*R*^2^ = 0.9900), supported by ANOVA. Artificial Neural Network (ANN) and Support Vector Machine (SVM) models were further employed to predict and validate the system performance, showing strong agreement with the experimental data. Overall, the combination of the developed photocatalyst with advanced modelling techniques offers a promising and sustainable strategy for removing persistent organic dyes from wastewater.

## Abstract 2

Ternary NiCrSe-chitosan microspheres exhibit efficient visible-light-driven photocatalytic degradation of Congo red. RSM, ANN and SVM modelling offer accurate optimisation and prediction, showing a promising strategy for sustainable water treatment.

## Graphical abstract

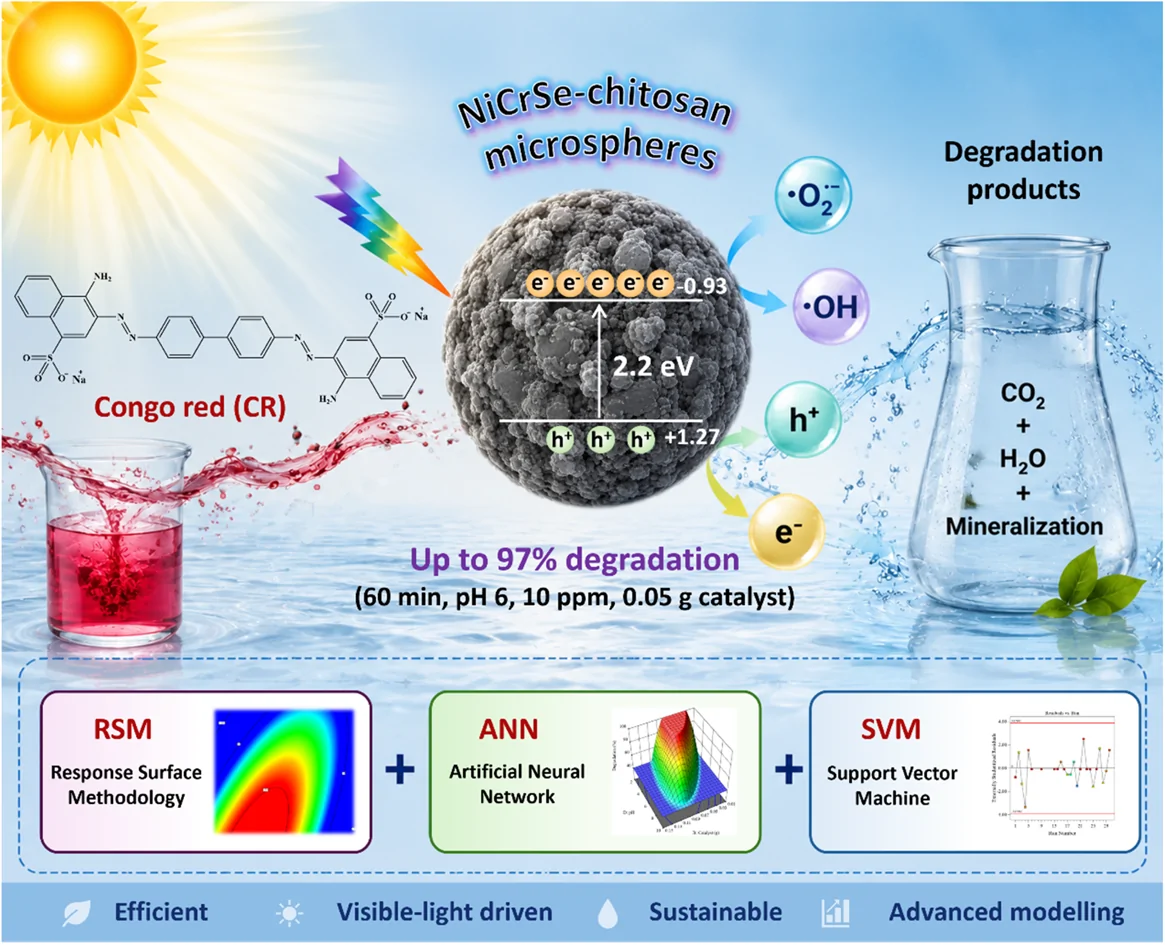

## 1. Introduction

The importance of clean water resources for the sustainable development of society is self-evident. Over the past few decades, rapid population growth and industrial development have increased the water crisis. Removing pollutants from wastewater is one of the most effective ways to access clean water. Organic dyes are widely used in printing, papermaking, textiles, plastics, leather tanning, cosmetics and other industries.^1,2^ Recent reports from the World Health Organization (WHO) indicate that approximately 844 million people globally still lack access to safe and basic drinking water.^3^ The remediation of contaminated wastewater has thus gained tremendous attention due to the adverse environmental effects of many emerging water pollutants (*e.g.*, heavy metals, organic pollutants, drug molecules, radionuclides, pharmaceuticals, personal care products *etc.*).^4^ The reactive azo dyes are water soluble and are washed out due to inefficient dyeing. Reports show discharge of about 2.8 × 10^5^ tons of textile dyes in the effluent produced by textile industries. Textile wastewater is typically characterized by high colour, high BOD (about 100–4000 mg L^−1^) and high COD (about 150–10 000 mg L^−1^), making it hazardous and toxic to flora, fauna and human health.^5^ Over 10 000 kinds of dyes with a total yearly production over 7 × 10^5^ tons worldwide are reported to be commercially available and approximately 10 % of dyestuffs are lost in the industrial effluents.^6^ Dye-containing wastewater is characterized by persistent, non-biodegradable-coloured compounds that are highly toxic to living organisms.^7–10^ Synthetic dyes can cause adverse health effects, including hypersensitivity, asthma, thyroid dysfunction, hyperactivity in children, and neurotoxicity in humans and animals.^11–13^

**Fig. 1:**
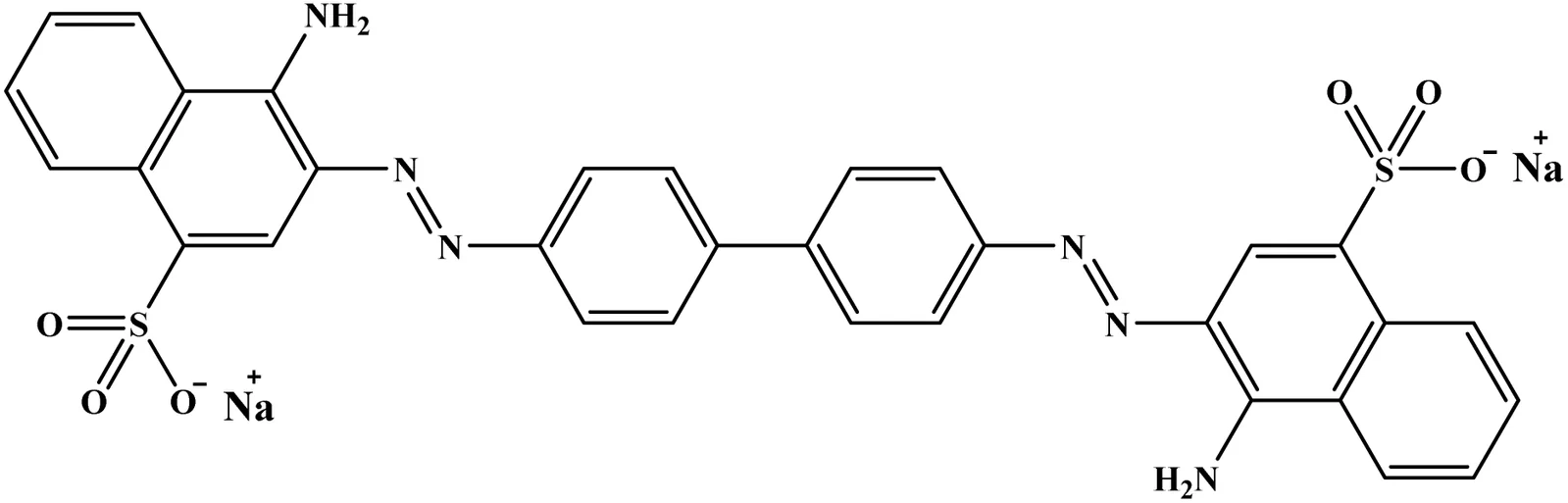
Molecular structure of Congo red dye.^23^

Colouring agents represent a major class of water pollutants that must be effectively removed from industrial effluents prior to discharge into aquatic systems to safeguard environmental and public health.^14^ Among these, Congo red (CR) is a synthetic diazo dye belonging to the family of anionic azo compounds, characterized by the presence of two azo (–N=N–) linkages connecting aromatic rings substituted with sulfonate groups, which impart high aqueous solubility and strong affinity toward fibrous substrates such as cellulose.^15^ These properties have led to its extensive application in textile processing, paper printing, rubber industries^16^ and as a biological staining agent in histopathology, particularly for amyloid detection.^17^ However, its complex conjugated aromatic structure renders it highly stable and resistant to natural biodegradation, enabling prolonged persistence in the environment.^18^ Upon release into water bodies, Congo red not only causes intense coloration that reduces light penetration and disrupts photosynthetic processes but also poses significant toxicological threats to aquatic organisms and human health.^19^ Under reductive conditions, it can degrade into hazardous aromatic amines, such as benzidine derivatives, which are known for their carcinogenic and mutagenic effects.^20,21^ Furthermore, recent studies have reported its involvement in oxidative stress induction, cytotoxicity, genotoxicity, and other adverse biological effects including skin irritation, respiratory disorders, and bioaccumulation potential.^20^ Due to these characteristics, Congo red is widely recognized as a model organic pollutant for evaluating advanced wastewater treatment techniques, particularly photocatalytic degradation approaches aimed at sustainable remediation.^22^ The molecular structure of Congo red is illustrated in (Fig. 1).

In this context, the synthesized (NiCrSe-CM) demonstrated significant photocatalytic efficiency for the degradation of Congo red under solar irradiation. The photocatalytic performance was systematically optimized by varying key operational parameters, including irradiation time, solution pH, catalyst dosage, and initial dye concentration. A comparative evaluation of the present photocatalyst with previously reported systems is summarized in SI (Table S1).

Various methods have been suggested for dye removal from water, including filtration,^24^ condensation,^25^ biodegradation^26–28^ coagulation.^29^ Flocculation,^30^ adsorption,^31^ membrane process and advanced oxidation process (AOP).^32–34^ Of all the recent technologies developed up to date, Advanced Oxidation Processes (AOPs) are the most efficient in removing these persistent organic pollutants from aqueous media and are considered eco-friendly treatment processes.^35^ The photocatalytic stimulated mechanism of this process is extensively explained in the SI (Section 2).^36^

Recent advances in the design of hybrid photocatalytic systems have increasingly focused on the integration of metal-based nanoparticles within biopolymeric matrices, particularly chitosan, due to its unique physicochemical properties and structural versatility.^37^ Chitosan offers abundant amino and hydroxyl functional groups that facilitate strong interactions with metal nanoparticles, enabling effective stabilization, prevention of aggregation, and enhanced dispersion of active sites. This incorporation not only improves the structural integrity of the catalyst but also promotes efficient charge separation and transfer, which are critical for photocatalytic performance. Furthermore, chitosan-based frameworks provide a porous and hydrophilic environment that enhances pollutant adsorption, thereby increasing the availability of target molecules at the catalyst surface.^38^ Recent studies have demonstrated that encapsulating metal selenide nanoparticles within chitosan matrices significantly enhances photocatalytic efficiency by synergistically combining adsorption capacity with catalytic activity, while also enabling facile recovery and reuse of the catalyst system.^39^

Among visible-light-active semiconductors, metal selenides have attracted growing attention as photocatalysts for dye degradation owing to their relatively narrow band gaps, large surface areas and abundant active sites, which favour efficient solar-light harvesting and charge generation.^40^ Depending on the number of metal centres, selenide photocatalysts range from binary to ternary and quaternary compositions, with multinary selenides offering improved charge separation and redox activity.^41^ A variety of selenide-based systems have been reported for the photocatalytic removal of organic dyes under visible or solar light, including molybdenum selenide, NbSe_2_, ternary ZnO/ZnSe/MoSe_2_ heterostructures, BiVO_4_/ZnSe composites and quaternary Fe–Bi–Zn selenides, achieving high efficiencies for dyes such as methylene blue and Congo red.^42–44^ In particular, immobilising metal selenide nanoparticles within chitosan matrices combines the catalytic activity of the selenide with the adsorption capacity and recoverability of the biopolymer support, enabling efficient and reusable photocatalysts for dye mineralisation.^45,46^ Building on earlier reports of ternary metal selenide-chitosan microspheres for the photocatalytic degradation of Congo red.^46,47^ The present study introduces a new ternary composition, nickel–chromium selenide (NiCrSe), immobilised in chitosan microspheres. The incorporation of chromium yields a distinct ternary selenide system, whose structural, optical and photocatalytic behaviour towards Congo red is characterised and evaluated under solar irradiation.

Beyond catalyst design, the optimisation of photocatalytic degradation increasingly relies on statistical and machine-learning tools. Response Surface Methodology (RSM) enables systematic modelling of the individual and interactive effects of operating parameters such as pH, catalyst dosage, dye concentration and irradiation time, while artificial-intelligence approaches including Artificial Neural Networks (ANN) and Support Vector Machines (SVM) capture the nonlinear relationships governing degradation efficiency and reduce reliance on time-consuming trial-and-error experimentation.^48,49^ The combined use of RSM with ANN/SVM modelling therefore provides a robust framework for predicting and optimising photocatalytic performance, as adopted in the present study.

In the present study, ternary metal selenide nanoparticles were synthesized *via* a co-precipitation approach and subsequently supported onto chitosan-based microspheres to develop an efficient and recoverable photocatalytic system. The structural, morphological, and compositional properties of the synthesized nanoparticles and the resulting composite photocatalyst were comprehensively characterized using Fourier Transform Infrared Spectroscopy (FTIR), X-ray Diffraction (XRD), Energy Dispersive X-ray Analysis (EDX), Scanning Electron Microscopy (SEM), and zeta potential measurements. The photocatalytic performance of the prepared material was evaluated through the degradation of Congo red dye under solar light irradiation. Furthermore, key operational parameters, including irradiation time, solution pH, catalyst loading, and initial dye concentration, were systematically optimized to achieve maximum degradation efficiency.

## 2. Experimental

### 2.1 Materials and method

The chemicals, including Congo red dye, selenium dioxide (SeO_2_), nickel chloride, chromium chloride, sodium hydroxide (NaOH), chitosan, acetic acid, ethanol, glutaraldehyde and sodium dodecyl sulfate (SDS) were supplied by Sigma-Aldrich and used without further purification. All the synthesis and experimental procedures employed deionized water.

### 2.2 Synthesis of NiCrSe-NPs and NiCrSe-chitosan microspheres

Nickel–chromium selenide nanoparticles were synthesized through the co-precipitation method.^11^ Equimolar solutions (0.1 M) of nickel chloride, chromium chloride, and 0.2 M solution of selenium dioxide were prepared separately in deionized water. Subsequently, the prepared solutions of selenium dioxide, nickel chloride, and chromium chloride were mixed together in a beaker. A 10 mL (1%) solution of sodium dodecyl sulfate (SDS) was added to the reaction mixture. The mixture's pH was then adjusted to a basic value (pH ≈ 11) by the dropwise addition of 2 M sodium hydroxide solution under continuous stirring. This resulted in the formation of NiCrSe-NP precipitates. The mixture was stirred for a further five hours at 85 °C to ensure complete precipitation, after which it was filtered and the precipitate thoroughly washed with deionized water. Finally, the product was dried and stored in a clean vessel for further use.^45^

Chitosan solution (1.75% w/v) was prepared by dissolving 1.75 g of chitosan in 100 mL of 2% acetic acid. 40 mL of this solution was mixed with 0.48 g of NiCrSe nanoparticles and stirred for 1 h to achieve uniform dispersion. The mixture was then added dropwise into NaOH solution (2 M) using a syringe, resulting in the formation of spherical NiCrSe-chitosan microspheres (NiCrSe-CM). The microspheres were filtered, washed with deionized water, dried and stored for further use.^46^

To crosslink the chitosan microspheres, 30 mL of ethanol was added to a 250 mL beaker. Under continuous stirring, 0.5 mL of glutaraldehyde was introduced into the beaker, and the reaction mixture was stirred for 30 min. Subsequently, an additional 0.5 mL of glutaraldehyde was added, and the solution was further stirred for another 30 min. Finally, 1 mL of glutaraldehyde was added, and the mixture was stirred for 1 h to ensure complete crosslinking. After completion of the reaction, the cross linked microspheres were separated by filtration, thoroughly washed, and subsequently dried for further use.^50^

### 2.3 Evaluating the photocatalytic degradation of Congo red dye

The photocatalytic performance of NiCrSe-CM was evaluated through the degradation of Congo red dye in an aqueous system in a simple batch configuration. The reaction was conducted in an open borosilicate glass beaker (working volume = 100 mL) containing the Congo red solution and the NiCrSe-CM photocatalyst. The suspension was kept under continuous magnetic stirring using a magnetic stir bar on a magnetic stirrer plate (heating function not used), ensuring uniform dispersion of the catalyst and effective contact between the catalyst surface and the dye molecules. All reactions were performed in a transparent reaction vessel under continuous stirring to ensure uniform dispersion of the photocatalyst and effective interaction between the catalyst surface and dye molecules. Prior to light exposure, the suspension was maintained in dark conditions for 30 min to establish adsorption–desorption equilibrium between the dye and the catalyst surface. Photocatalytic process was carried out with (0.01 to 0.15 g) of catalyst dose, (10–90 ppm) of dye concentration, pH (2, 4, 6, 7, 8, 10) under constant stirring at 28–45 °C in solar light irradiation. All photocatalytic runs were carried out under natural sunlight (longitude 71°E, latitude 34°N) during the 12:00–14:30 window, at an average temperature of 35.5 °C and relative humidity of 38%. The irradiation (contact) time was varied from 10 to 60 min as a process variable (Section 3.8.1), with samples withdrawn at fixed intervals and analysed by UV-Vis spectrophotometry. The solar intensity was not recorded during individual runs; however, under the clear-sky midday conditions of these experiments, the global horizontal irradiance at this location (∼34°N) is typically on the order of 1000 W m^−2^, indicating that the reactions proceeded under near-peak solar flux. The mixture was centrifuged for 15 min to remove the catalyst.^51^ The photocatalytic efficiency was determined by measuring the absorbance of Congo red solution before and after solar irradiation using UV/Vis double-beam spectrophotometer UV-1800 (ENG 240 V Shimadzu). The percentage degradation was determined by using eqn (1).1
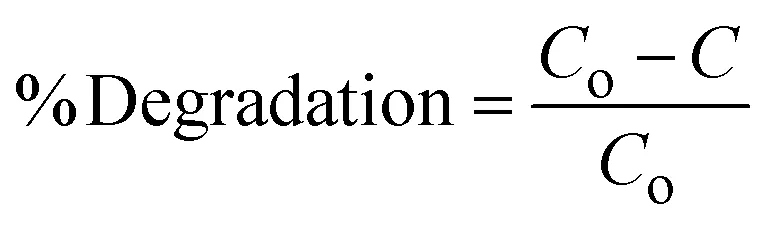
where *C*_o_ denotes initial absorbance, and *C* represents the final absorbance.^52,53^

### 2.4 Characterization

UV-visible diffuse reflectance spectroscopy (UV-Vis DRS) was used to analyze the optical behaviour of the NiCrSe-NPs. The absorbance spectrum was recorded within the 200–800 nm wavelength range. To find out the optical band gap, Tauc method was applied where a graph was plotted between absorption coefficient (*α*) and photon energy (*hv*). The photocatalyst's band gap was calculated from a Tauc plot using the Tauc equation in (eqn (2)–(4)).

2 (*αhv*)^*n*^ = *A*(*hν* − *E*_g_)

3
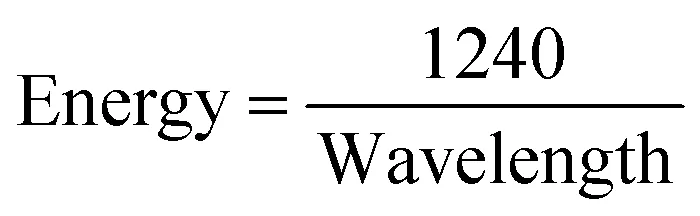

4 (*αhν*) = (2.303 × abs × energy)^2^ (eV^−2^ cm^−2^)

Scanning Electron Microscopy (SEM) was employed to examine the surface morphology, particle size, and structural features of the synthesised NiCrSe nanoparticles and their corresponding chitosan-based composite microspheres using a JEOL JSM-5910 SEM (Japan). In addition, Energy Dispersive X-ray (EDX) spectroscopy was utilized to determine the elemental composition and purity of the synthesized NiCrSe nanoparticles. The EDX measurements were performed using an INC-200 EDX spectrometer (Oxford Instruments, UK).

Fourier Transform Infrared (FTIR) spectroscopy was employed to investigate the chemical structure and functional groups present in the synthesized NiCrSe-CM and NiCrSe-NPs. The analysis was performed over a broad wavenumber range using an Attenuated Total Reflectance (ATR) system (Thermo Electron Corporation, USA). The obtained spectra confirmed the successful formation of the composite material.

X-ray Diffraction (XRD) analysis was conducted to elucidate the crystalline structure and phase composition of the synthesized NiCrSe-NPs. The measurements were performed using a JEOL XRD instrument (JDX-9C, Japan) under ambient conditions. Diffraction patterns were recorded over an appropriate range of diffraction angles (2*θ*) using Cu Kα radiation with a characteristic wavelength of 0.15406 nm. The resulting XRD patterns were analysed to identify crystalline phases, assess structural purity, and estimate crystallinity of the prepared nanoparticles. The average crystallite size of the synthesized nanoparticles was estimated from the XRD data using the Scherrer eqn (5):5
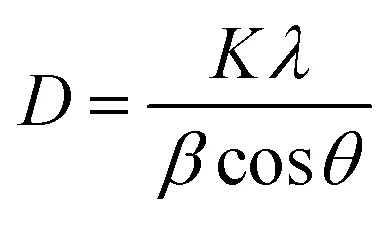
where *D* represents the crystallite size, *k* is Scherrer's constant (0.9), *λ* corresponds to the wavelength of the X-ray radiation, *β* denotes the full width at half maximum (FWHM) of the diffraction peak, and *θ* is the Bragg diffraction angle.

### 2.5 RSM modelling

Response Surface Methodology (RSM) was employed to relate the process variables to the photocatalytic degradation efficiency of Congo red and to identify the optimum conditions. Four independent variables solution pH, catalyst dosage, initial dye concentration, and irradiation time were studied over defined ranges using a central composite design (*N* = 30 runs); Table 4, and the response (% degradation) was fitted to a second-order polynomial model (eqn (6)):

6 *Y* = *β*_0_ + ∑*β*_*i*_*X*_*i*_ + ∑*β*_*ii*_*X*_*i*_^2^ + ∑*β*_*ij*_*X*_*i*_*X*_*j*_

where *Y* is the predicted response, *β*_0_ the intercept, and *β*_*i*_, *β*_*ii*_ and *β*_*ij*_ the linear, quadratic and interaction coefficients of the coded variables *X*_*i*_. Model adequacy was assessed by ANOVA (*F*-test, *p*-values at 95% confidence, and lack-of-fit), the coefficients of determination (*R*^2^, adjusted *R*^2^, predicted *R*^2^), adequate precision and coefficient of variation; the relative contribution of each factor was estimated from its sum of squares. The fitted model was visualised using perturbation, 2D contour and 3D response-surface plots, and the predicted optimum was verified experimentally. All analyses were performed in Design–Expert.^46^

#### 2.5.1 ANN model

An artificial neural network (ANN) was used to predict the degradation efficiency from the same four variables.^54^ A feed-forward network (input–hidden–output layers) was trained on the RSM dataset Table 4 after preprocessing and slight augmentation, with random partitioning into training, testing and validation subsets; the architecture (number of hidden neurons and transfer functions) was optimised to minimise prediction error. Performance was evaluated using the mean squared error (MSE) and *R*^2^ (eqn (7) and (8)). The ANN reproduced the nonlinear behaviour well, complementing the RSM optimisation:7
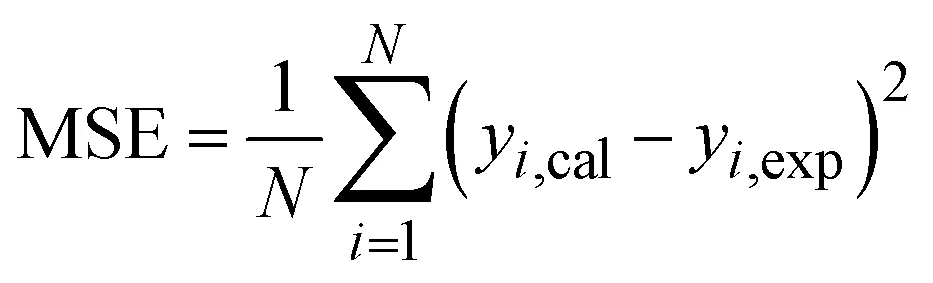
8
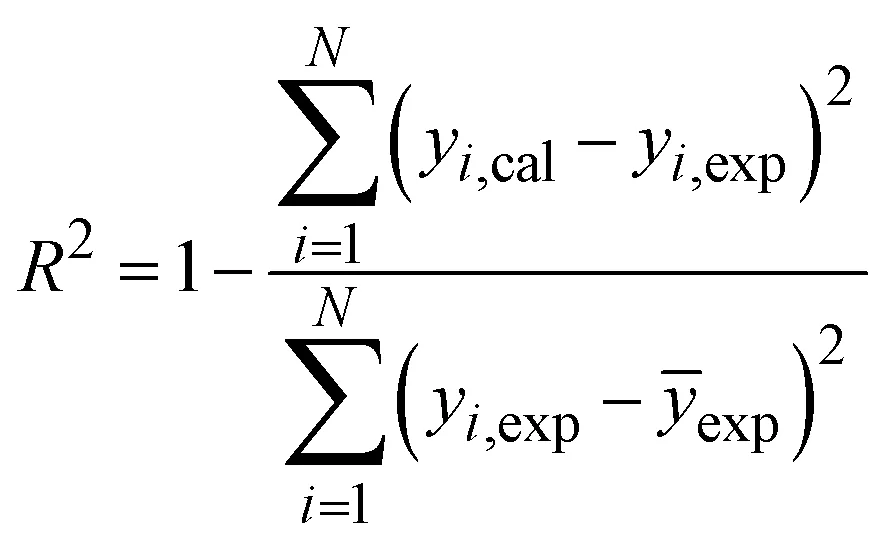
where *N* represents the total number of experimental data points, *y*_exp_ and *y*_cal_ denote the experimental and predicted values, respectively, and *ȳ* exp is the mean of experimental responses. The ANN model demonstrated strong capability in capturing the nonlinear interactions among process variables and provided reliable predictions for CR degradation efficiency, thereby complementing the RSM-based optimization approach.

#### 2.5.2 SVM modelling

A support-vector-machine (SVM) regression model was also developed from the same dataset Table 4. A radial basis function (RBF) kernel was used, and the hyperparameters regularisation parameter *C*, kernel coefficient *γ*, and insensitive loss *ε* were tuned to balance accuracy and generalisation. The regression function is given in eqn (9):9
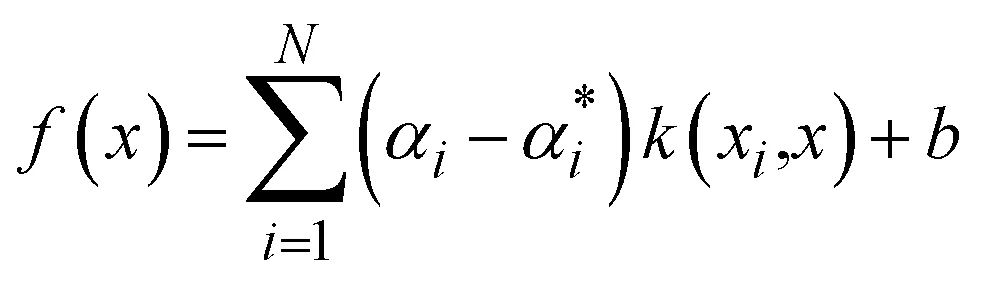
where *α*_*i*_ and 
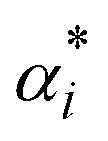
 are Lagrange multipliers, *k*(*x*_*i*_, *x*) the kernel function, and *b* the bias. Predicted and experimental efficiencies showed strong agreement, confirming the robustness of the model.^55^

## 3. Results and discussion

### 3.1 Fourier transform infrared (FTIR)

**Fig. 2:**
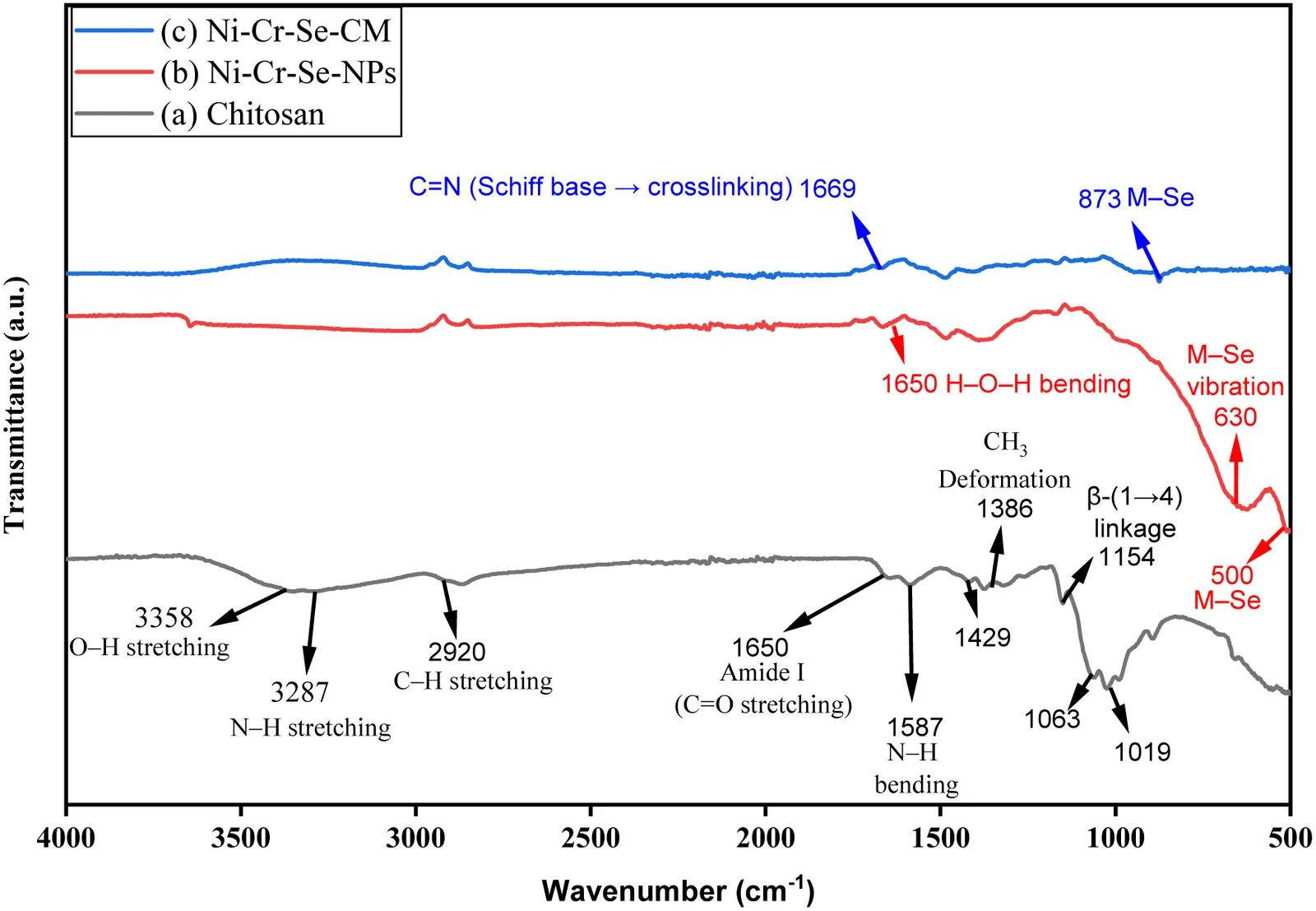
FTIR spectra of (a) pure chitosan, (b) NiCrSe nanoparticles, and (c) NiCrSe-chitosan microspheres, showing characteristic functional groups and metal–selenium bonding.

FTIR analysis was carried out in the range of 4000–400 cm^−1^ to comparatively evaluate pure chitosan (Fig. 2a), NiCrSe nanoparticles (Fig. 2b), and the NiCrSe-chitosan composite (Fig. 2c), thereby confirming the successful formation of the photocatalyst system.

The spectra of all samples exhibit common broad bands in the higher wavenumber region around 3361–3628 cm^−1^ and ∼1661 cm^−1^, which are attributed to O–H stretching and bending vibrations, respectively, indicating the presence of surface hydroxyl groups and adsorbed moisture. In the chitosan spectrum, characteristic features such as 2980–2868 cm^−1^ (C–H stretching), 1650 cm^−1^ (C=O stretching), 1587 cm^−1^ (N–H bending), and the region 1200–800 cm^−1^ (pyranosidic ring vibrations including C–O–C and β-(1 → 4) glycosidic linkages at 1154 cm^−1^) confirm its polysaccharide structure.^11,47,50,53,56^ Upon formation of NiCrSe nanoparticles, new distinct peaks emerge at 500 cm^−1^, 630 cm^−1^, corresponding to metal–selenium (M–Se) stretching vibrations, verifying the successful synthesis of ternary metal selenide nanostructures.^57,58^ These M–Se vibrational bands are retained in the composite spectrum, demonstrating effective incorporation of nanoparticles within the chitosan matrix.^47,59^ Additionally, the appearance of a new band at 1674 cm^−1^ in the composite is attributed to C=N stretching, indicating Schiff base formation due to cross-linking, which further confirms structural modification and stabilization of the microspheres.

### 3.2 Scanning electron microscopy (SEM) and energy dispersive X-ray analysis (EDX)

**Fig. 3:**
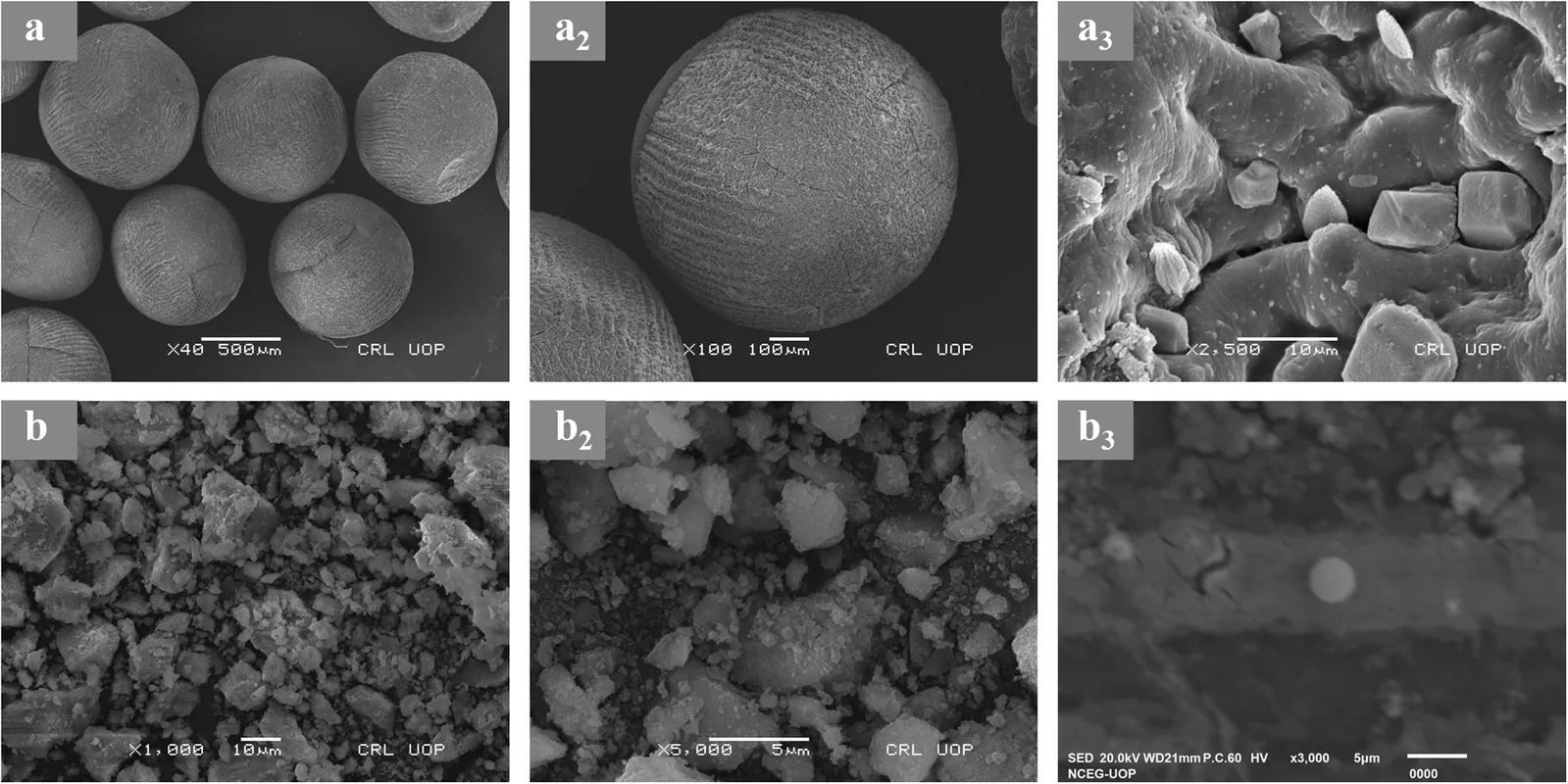
Shows SEM analysis of (a) NiCrSe-NPs and (b) NiCrSe-CM microspheres.

SEM analysis was conducted to examine the morphology and particle size of the synthesized NiCrSe-CM (Fig. 3a) and NiCrSe-NPs (Fig. 3b). The average particle size of the NiCrSe-NPs was approximately 76 nm, measured using ImageJ software. The nanoscale size provides an increased surface area and more active sites, which enhances the photocatalytic degradation of organic dyes. SEM analysis also revealed that the NiCrSe-CM composite consists of uniformly distributed, spherical chitosan microspheres with a highly porous surface morphology (average size ∼898 µm), as shown in Fig. 3a. This porous architecture enhances dye adsorption and facilitates effective interaction between the catalyst and Congo red molecules, thereby promoting improved photocatalytic degradation under sunlight irradiation.^47^

**Fig. 4:**
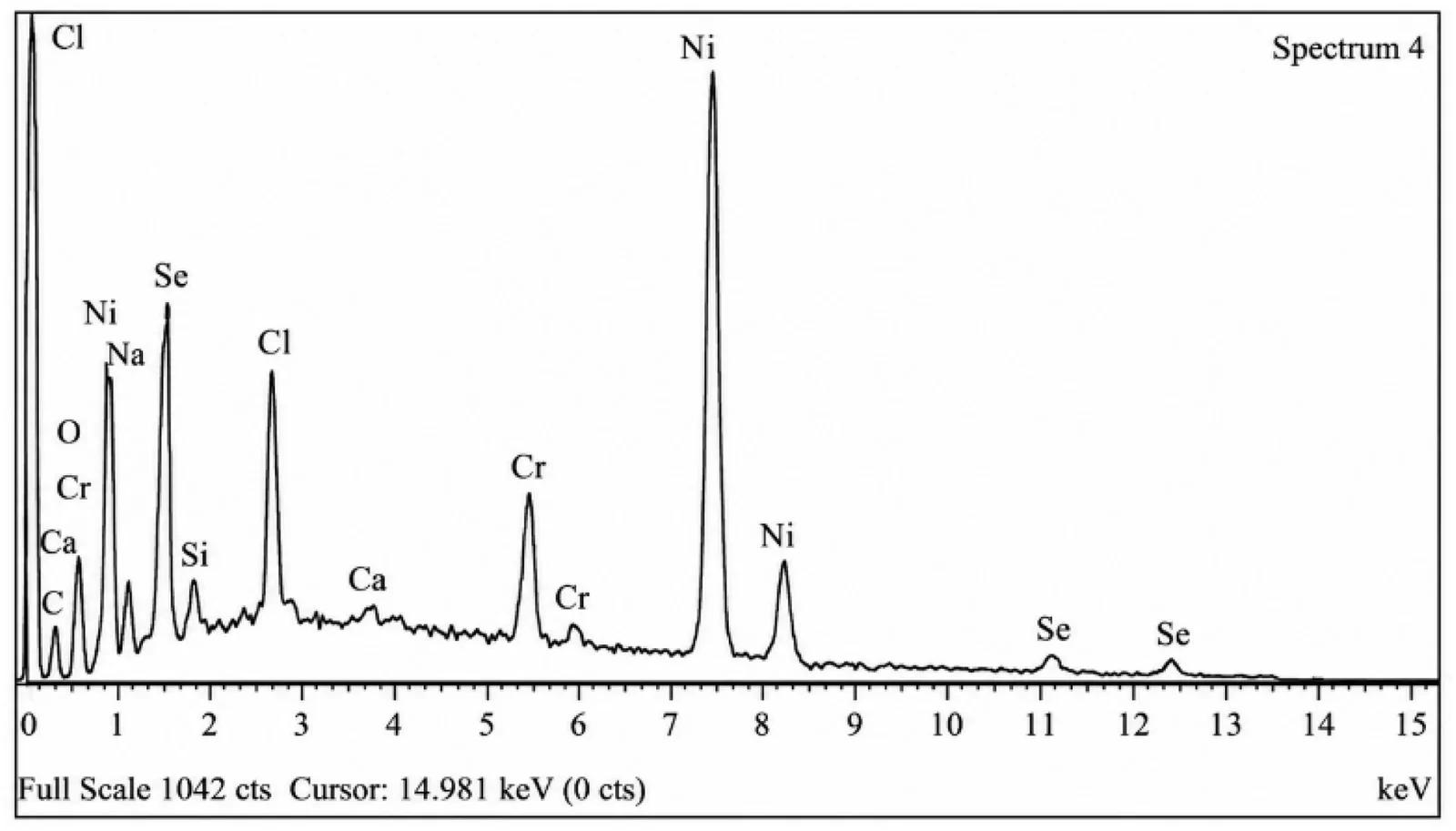
EDX spectrum and elemental mapping of NiCrSe nanoparticles.

Elemental composition of the synthesized metal selenide nanoparticles (NiCrSe-NPs) were confirmed through EDX analysis, as illustrated in Fig. 4. The obtained EDX spectrum exhibited distinct peaks for nickel (Ni), chromium (Cr), and selenium (Se) confirming the presence of the desired elements. Additionally, minor signals for sodium (Na), oxygen (O) and carbon (C), likely originating from residual SDS, were also observed, supporting the successful formation of NiCrSe-NPs.

### 3.3 X-ray diffraction analysis

**Fig. 5:**
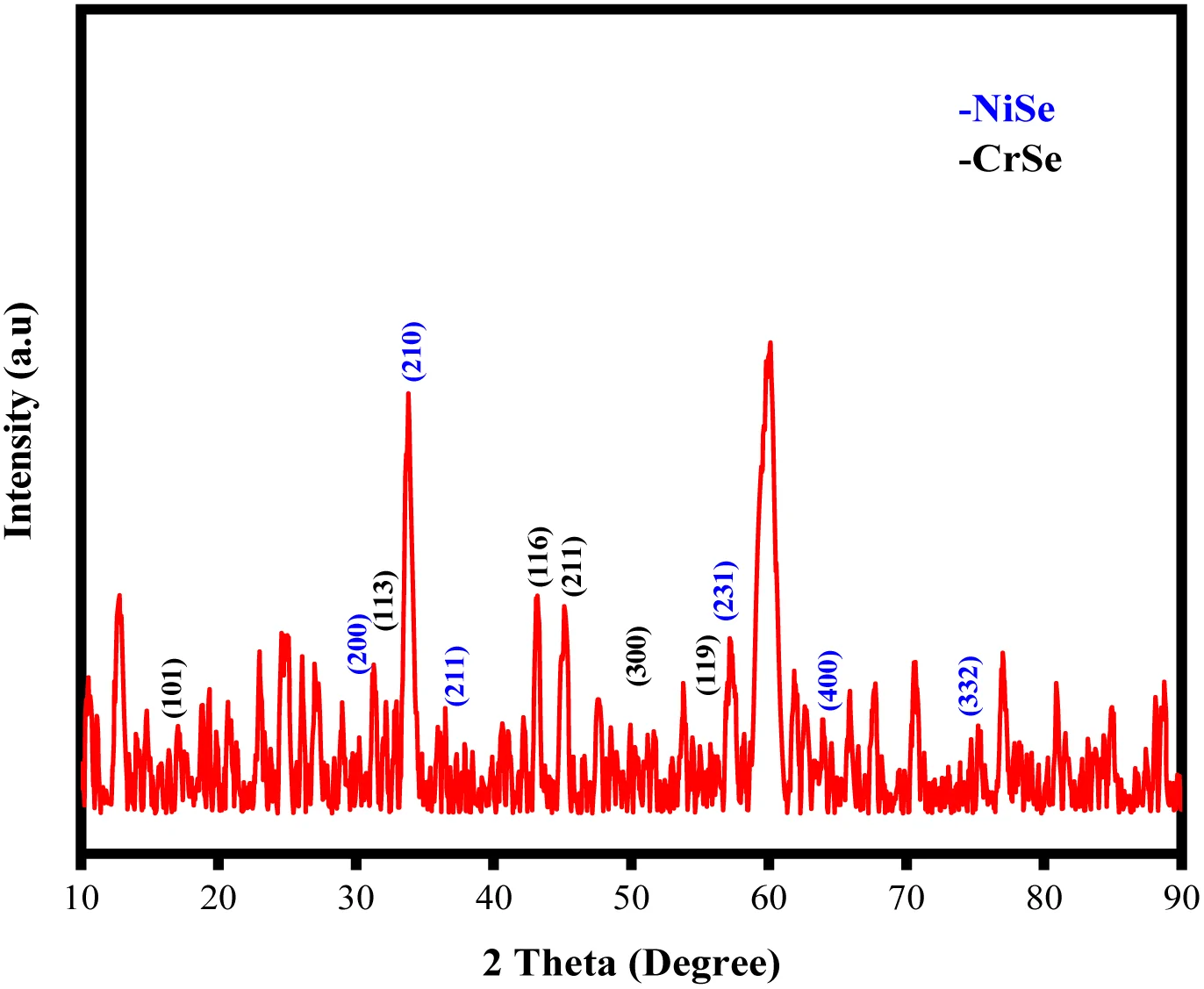
X-ray diffraction (XRD) showing the characteristic peaks of NiSe and CrSe.

X-ray diffraction (XRD) analysis was used to study the nanoparticle crystal structure, as depicted in Fig. 5. The diffraction peaks corresponding to NiSe (JCPDS card no. 41-1495) appear at 30.2°, 33.9°, 37.1°, 57.5°, 63.8° and 74.5° which can be indexed to the (200), (210), (211), (321), (400) and (332) crystallographic planes, respectively.^60–62^ In addition, the peaks related to CrSe (JCPDS card no. 40-1403, ICDD database) are observed at 16.5°, 32.5°, 42.5°, 45.5°, 50.6°, and 56.1°, corresponding to the (101), (113), (116), (211), (300), and (119) planes, respectively. The relatively higher value of intensity of peaks of NiSe can be explained by the higher concentration of nickel in the NiCrSe system.

**Fig. 6:**
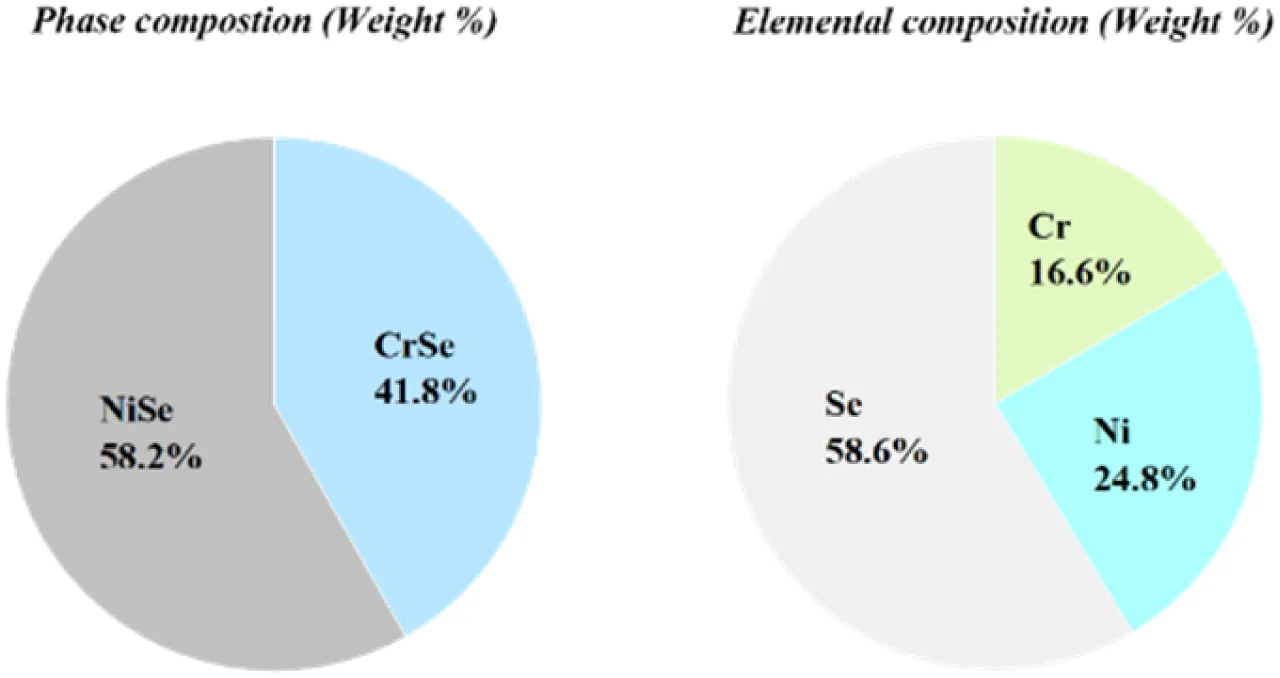
Phase and elemental composition of NiCrSe nanoparticles.

Moreover, the steep diffraction peak intensity increase indicates that the effective selenization of Ni and Cr had improved the structural integrity and the crystallinity of the catalyst. The crystallite size of NiCrSe-NPs was calculated to be approximately 35 nm using the Debye–Scherrer equation as explained in Characterization. The analysis of the prominent diffraction peaks indicated that the synthesized NiCrSe-NPs possess nanoscale crystallite dimensions, confirming the successful formation of a finely crystalline nanostructured material.^11,63,64^ The phase composition in Fig. 6 has been calculated from XRD data given above.

### 3.4 Zeta potential analysis

**Fig. 7:**
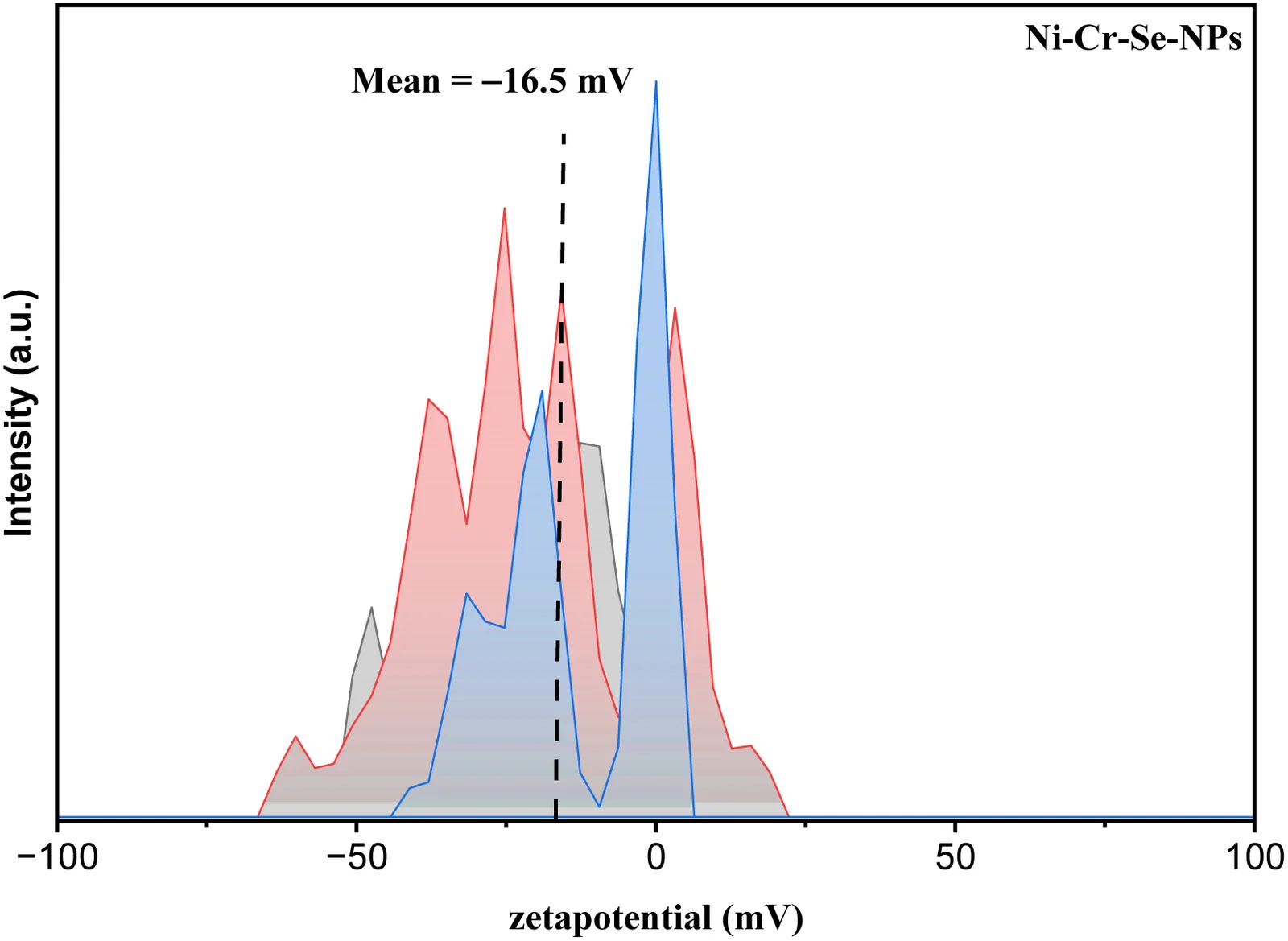
Zeta-potential (surface charge) distribution of NiCrSe nanoparticles.

**Fig. 8:**
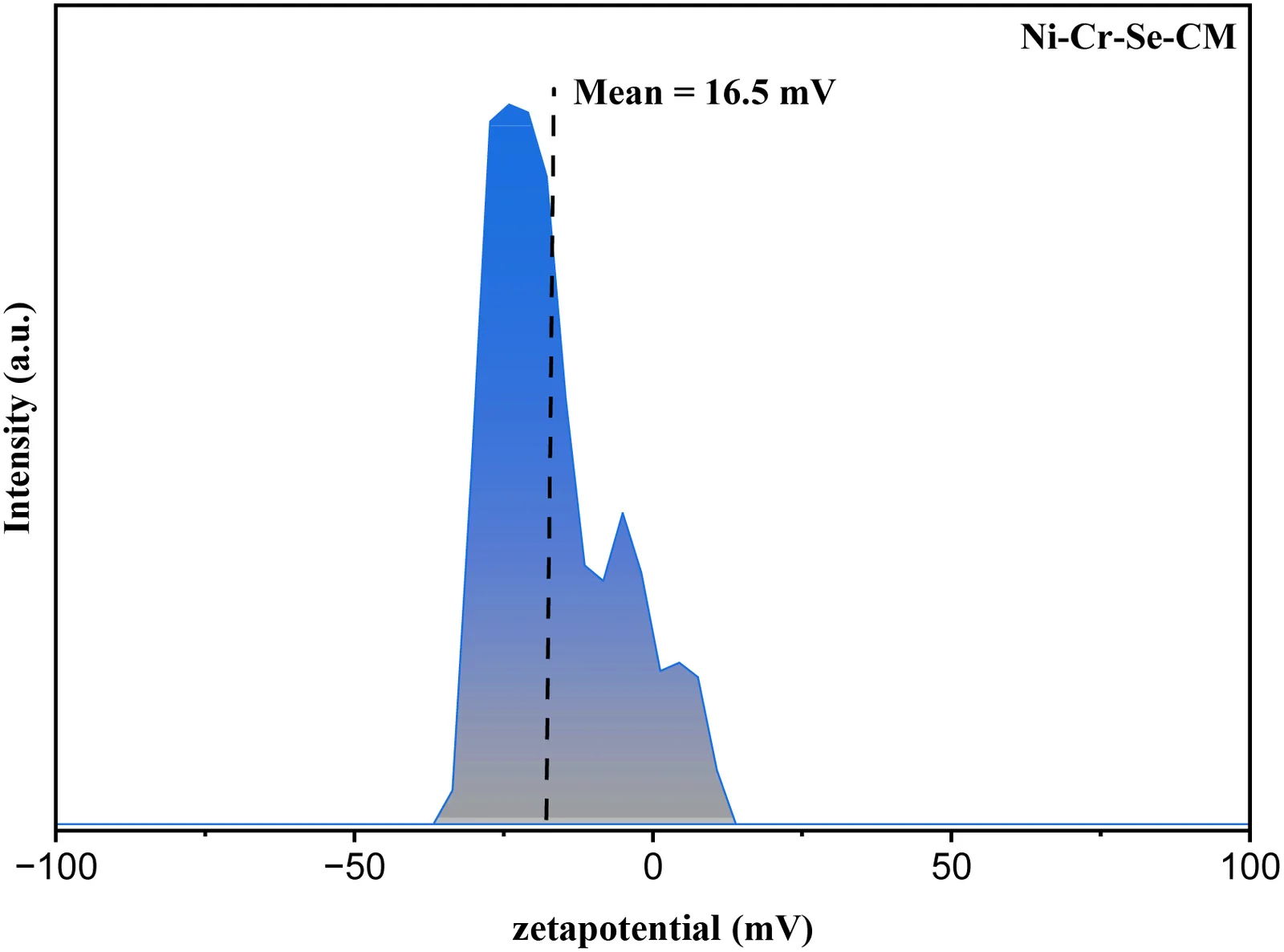
Zeta-potential (surface charge) distribution of NiCrSe-chitosan microspheres.

Zeta potential reflects the surface charge and colloidal stability of nanoparticles, with higher absolute values indicating greater resistance to aggregation.^65–67^ The zeta potentials of NiCrSe-NPs and NiCrSe-CM are shown in Fig. 7 and 8. The mean values are −16.5 mV for the NPs and −16.5 mV for the microspheres, indicating good colloidal quality and comparatively good stability, as summarized in Table 1. The obtained value indicates that, under acidic conditions, the photocatalyst facilitates the adsorption of the anionic dye (Congo red), leading to enhanced photocatalytic efficiency.^68^

**Table 1:** Zeta-potential analysis of NiCrSe-NPs and NiCrSe-CM (mean and peak potentials, conductivity, and quality factor)

Sample designation	Mean zeta potential (mV)	Peak 1 (mV)	Peak 2 (mV)	Conductivity (mS cm^−1^)	Quality factor
NiCrSe-NPs	−16.58	−45.44	−31.88	0.412	1.828
NiCrSe-CM	−16.50	−20.97	−4.06	0.393	1.586

### 3.5 UV-DRS spectroscopy

**Fig. 9:**
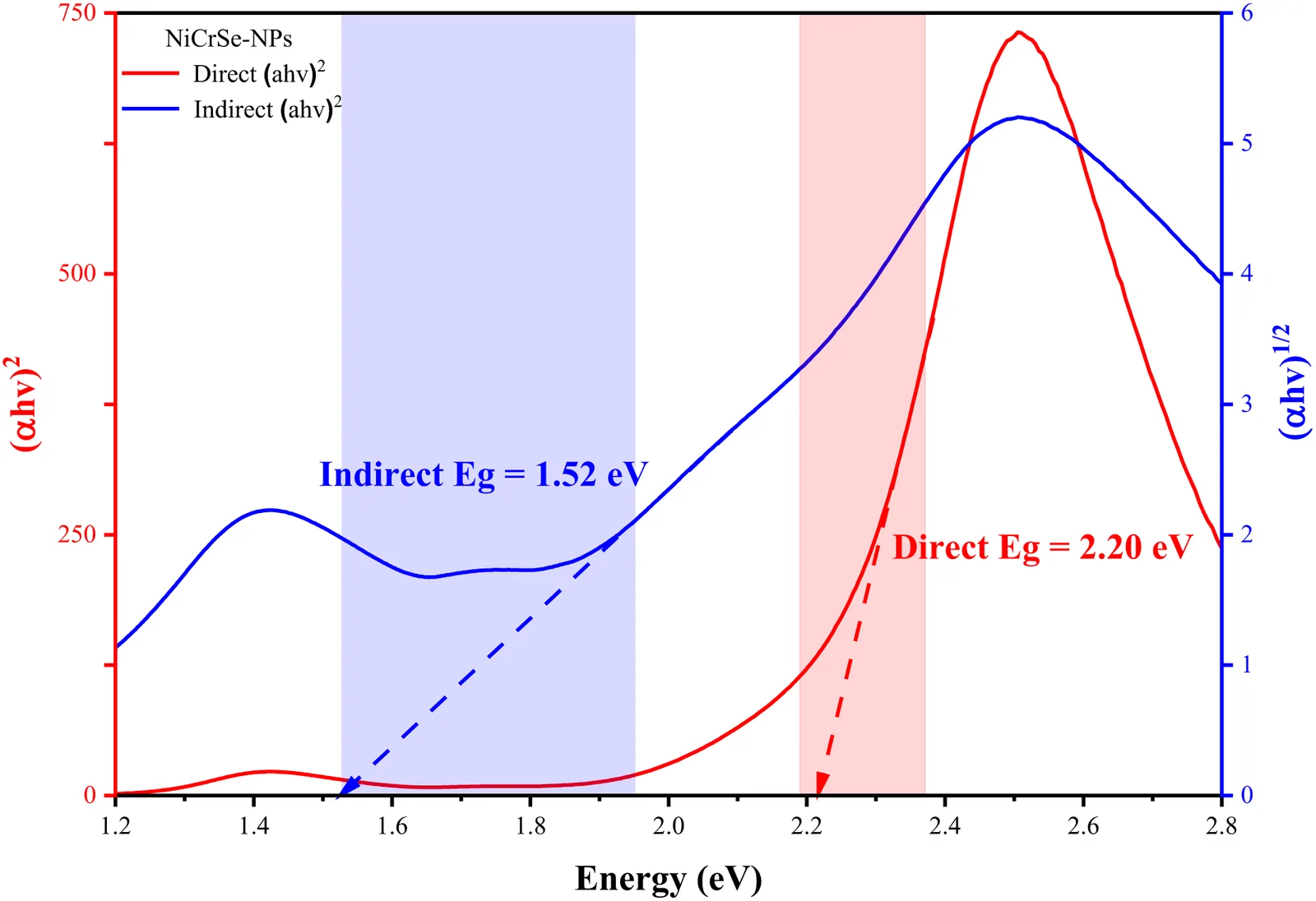
UV/visible absorption spectrum and corresponding Tauc plot for NiCrSe nanoparticles. NiCrSe-NPs.

The optical properties of the synthesised NiCrSe-NPs were investigated by UV-visible diffuse-reflectance spectroscopy, and the band gap was estimated using the Tauc method for both direct and indirect allowed transitions. Extrapolating the linear region of the (*αhν*)^2^*versus hν* plot gave a direct band gap of ≈2.2 eV, while the (*αhν*)^½^*versus hν* plot gave an indirect band gap of ≈1.5 eV (Fig. 9).^45,47,53,57^ The extracted value is sensitive to the chosen linear-fit range, resulting in an uncertainty of about ±0.2 eV. The direct band gap of ≈2.2 eV corresponds to an absorption edge at ≈564 nm in the visible region, and this visible-light absorption promotes the generation of electron–hole pairs, facilitating the formation of reactive species and contributing to the photocatalytic degradation efficiency.^69^

### 3.6 Mott–Schottky analysis

**Fig. 10:**
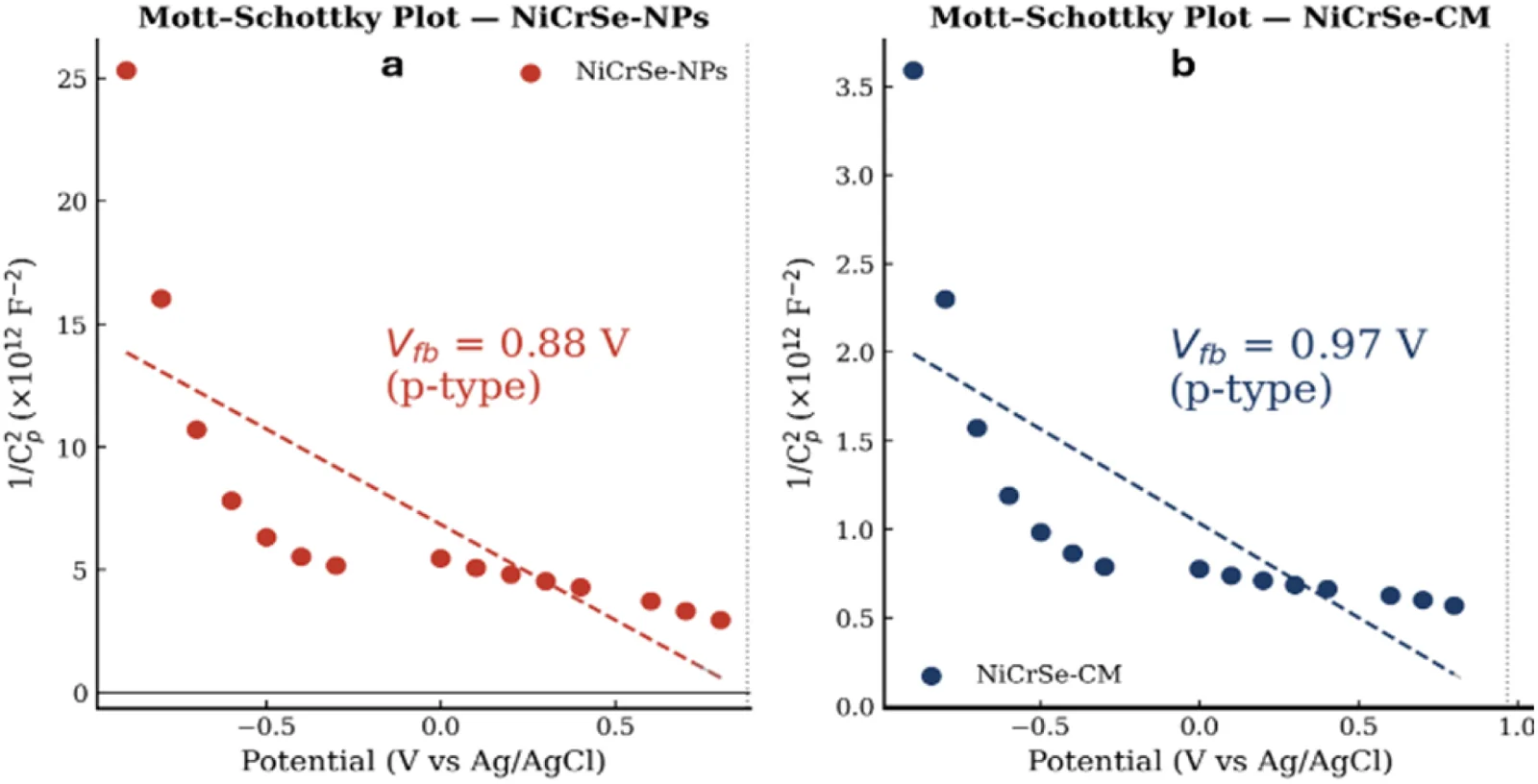
Mott–Schottky plots (1/*C*^2^*vs.* potential *vs.* Ag/AgCl) for (a) NiCrSe-NPs and (b) NiCrSe-CM measured at 1000 Hz in 0.1 M Na_2_SO_4_ electrolyte.

The Mott–Schottky analysis confirmed that both NiCrSe-NPs and NiCrSe-CM exhibit p-type semiconductor behaviour, as indicated by the negative slope of the 1/*C*^2^*versus* potential plots Fig. 10.

The negative slope of the 1/*C*^2^*versus* potential plots for both samples (Fig. 10) confirms p-type semiconductor behaviour for both NiCrSe-NPs and NiCrSe-CM. The flat-band potentials were determined to be +0.88 V and +0.97 V *vs.* Ag/AgCl (+1.08 V and +1.17 V *vs.* NHE) for NiCrSe-NPs and NiCrSe-CM, respectively. From the flat-band potential of NiCrSe-CM and the optical band gap of 2.2 eV, the valence band edge was estimated at *E*_VB_ ≈ +1.27 V *vs.* NHE and the conduction band edge at *E*_CB_ ≈ −0.93 V *vs.* NHE. Since *E*_CB_ (−0.93 V) is more negative than the O_2_/˙O_2_^−^ redox couple (−0.33 V *vs.* NHE), photogenerated electrons can readily reduce dissolved oxygen to superoxide radicals (˙O_2_^−^). As *E*_VB_ (+1.27 V) is less positive than the ˙OH/OH^−^ couple (+1.99 V *vs.* NHE), direct oxidation of water by valence-band holes is thermodynamically unfavourable; instead, photogenerated holes act as direct oxidants towards the adsorbed Congo red molecules. The p-type character and the favourable band-edge positions collectively explain the strong photocatalytic activity of NiCrSe-CM under solar irradiation. The experimentally determined flat-band potentials and the corresponding conduction and valence band edge positions for both NiCrSe-NPs and NiCrSe-CM are summarised in Table 2.

**Table 2:** Band-edge positions of NiCrSe-NPs and NiCrSe-CM from Mott–Schottky analysis (*E*_g_ = 2.2 eV, measured at 1000 Hz in 0.1 M Na_2_SO_4_)

Sample designation	Semiconductor type	*V*_fb_ (V *vs.* Ag/AgCl)	*V*_fb_ (V *vs.* NHE)	*E*_CB_ (V *vs.* NHE)	*E*_VB_ (V *vs.* NHE)
NiCrSe-NPs	p-Type	+0.88	+1.08	−1.02	+1.18
NiCrSe-CM	p-Type	+0.97	+1.17	−0.93	+1.27

### 3.7 Photoluminescence (PL) spectra

**Fig. 11:**
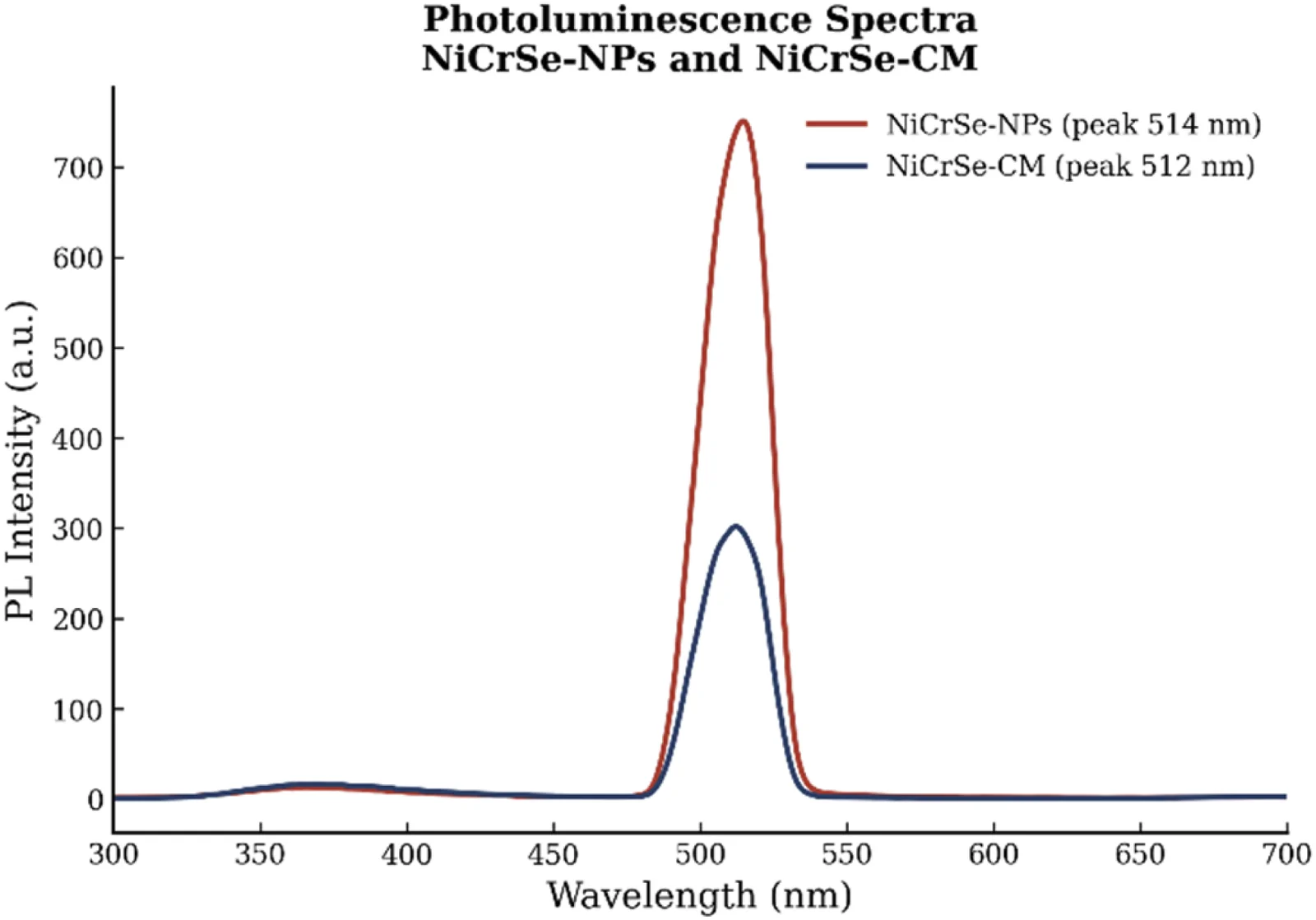
Photoluminescence spectra of NiCrSe-NPs and NiCrSe-CM recorded under excitation at 250 nm, showing suppressed recombination in the composite.

The charge-carrier recombination behaviour of NiCrSe-NPs and NiCrSe-CM was investigated by photoluminescence (PL) spectroscopy under excitation at 250 nm Fig. 11.

Both samples exhibited a prominent emission peak at approximately 512–514 nm, consistent with band-edge emission from the NiCrSe selenide system. Importantly, the PL intensity of NiCrSe-CM was approximately 2.5-fold lower than that of the bare NiCrSe-NPs. Since PL emission arises from the radiative recombination of photogenerated electron–hole pairs, a lower intensity indicates suppressed recombination and more efficient charge separation. The significant quenching of PL intensity upon immobilisation in the chitosan microsphere matrix confirms that the biopolymer support promotes charge separation, allowing a greater proportion of photogenerated carriers to migrate to the catalyst surface and participate in the degradation of Congo red. This result is consistent with the superior photocatalytic performance of NiCrSe-CM compared to NiCrSe-NPs and supports the proposed mechanism based on the Mott–Schottky band-edge positions.

### 3.8 Photocatalytic performance of NiCrSe-CM

#### 3.8.1 Influence of light irradiation time

**Fig. 12:**
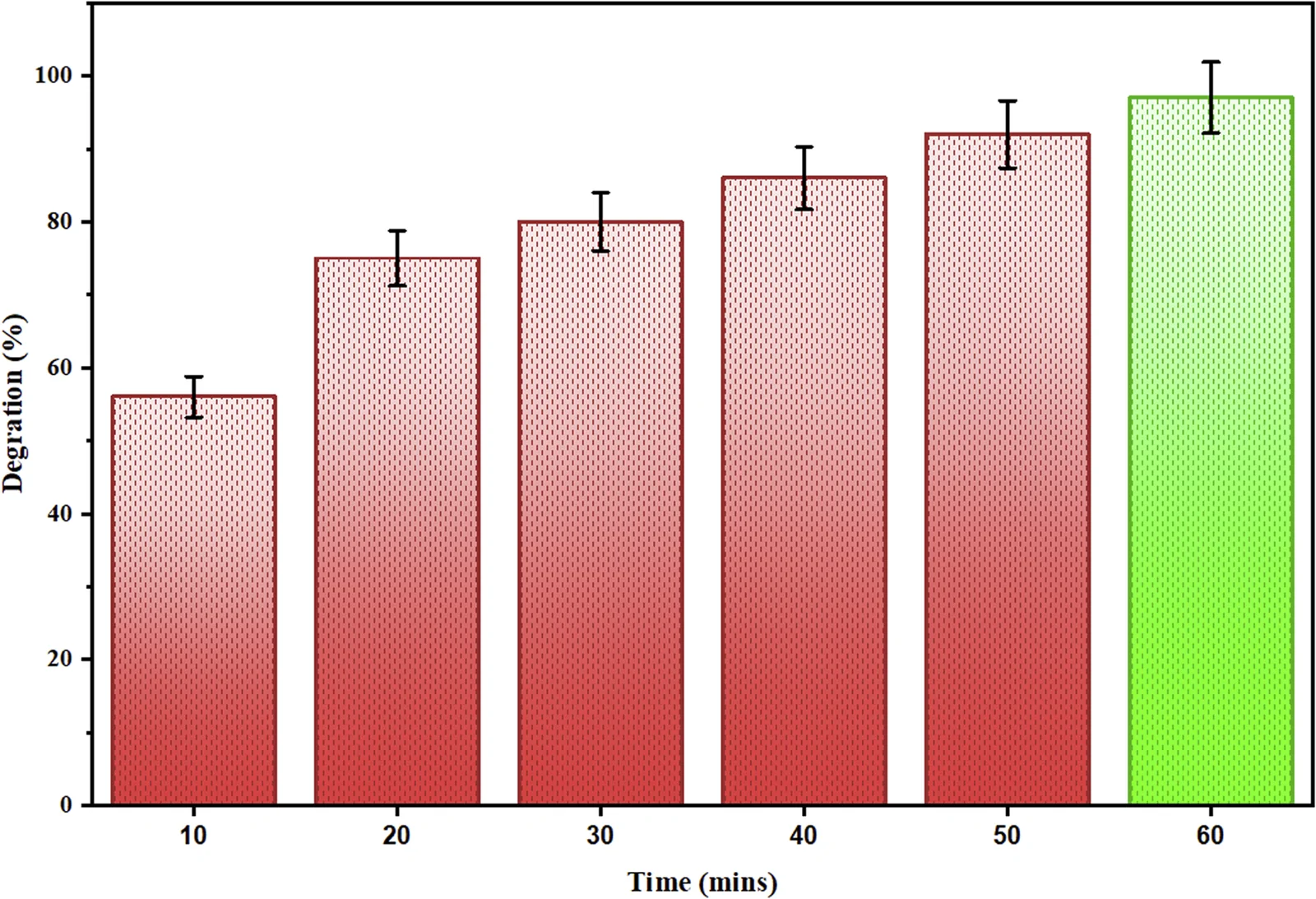
Effect of irradiation time on the photocatalytic degradation of Congo red.

The influence of contact duration (10–60 min) on the photocatalytic degradation of Congo red (CR) dye was investigated using NiCrSe-CM as the photocatalyst, as shown in Fig. 12, under reaction conditions, including CR concentration of 20 ppm, catalyst dosage of 0.1 g, and neutral pH.

The results obtained indicated significant improvements in degradation efficiency in the course of irradiation time, especially during the initial stages of the reaction time. This increase can be attributed to the increased number of dye molecules available to photocatalytic degradation in the initial stages of the reaction. However, as the reaction continues, the concentration of both the dye and reactive radicals decreases, and hence, the degradation rate reveals a gradual decrease. It was observed that the NiCrSe-CM demonstrated excellent photocatalytic performance to degrade CR dye to 97% under sunlight irradiation within 60 min.^70–72^

**Fig. 13:**
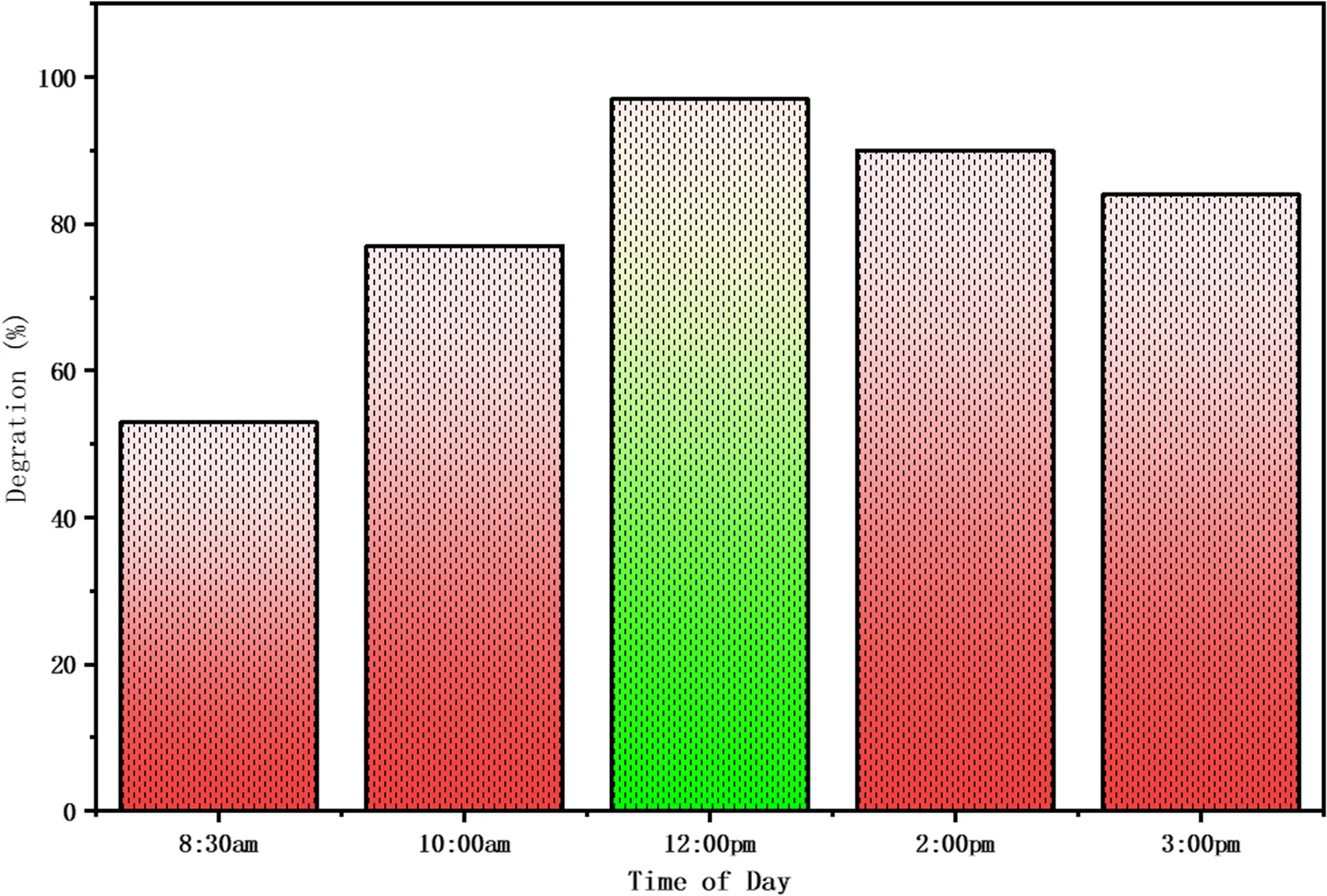
Effect of time of day on the photocatalytic degradation of Congo red by NiCrSe-CM under natural solar irradiation (Peshawar, 34°N, 71°E). Conditions: 0.05 g catalyst, 10 ppm dye, pH 6, 60 min irradiation.

The effect of the time of day on photocatalytic degradation was investigated by conducting experiments at five different solar irradiation periods under otherwise identical optimum conditions Fig. 13.

The degradation efficiency increased progressively from 53% at 8:30 am to a maximum of 97% at 12:00 pm, after which it declined to 90% at 2:00 pm and 84% at 3:00 pm. This trend closely follows the diurnal variation of solar irradiance at Peshawar (34°N, 71°E), where solar intensity rises through the morning, reaches its peak around solar noon, and gradually decreases in the afternoon. The highest degradation at midday is attributed to the maximum photon flux available to excite the NiCrSe-CM photocatalyst, generating the greatest number of electron–hole pairs and consequently the highest concentration of reactive species. The decrease in the afternoon reflects the declining solar angle and reduced irradiance. Importantly, the catalyst maintained high efficiency (≥84%) across all afternoon time points, demonstrating that NiCrSe-CM remains effective throughout the main daylight hours and is therefore suitable for practical solar-driven wastewater treatment applications without requiring precise timing of operation.

**Fig. 14:**
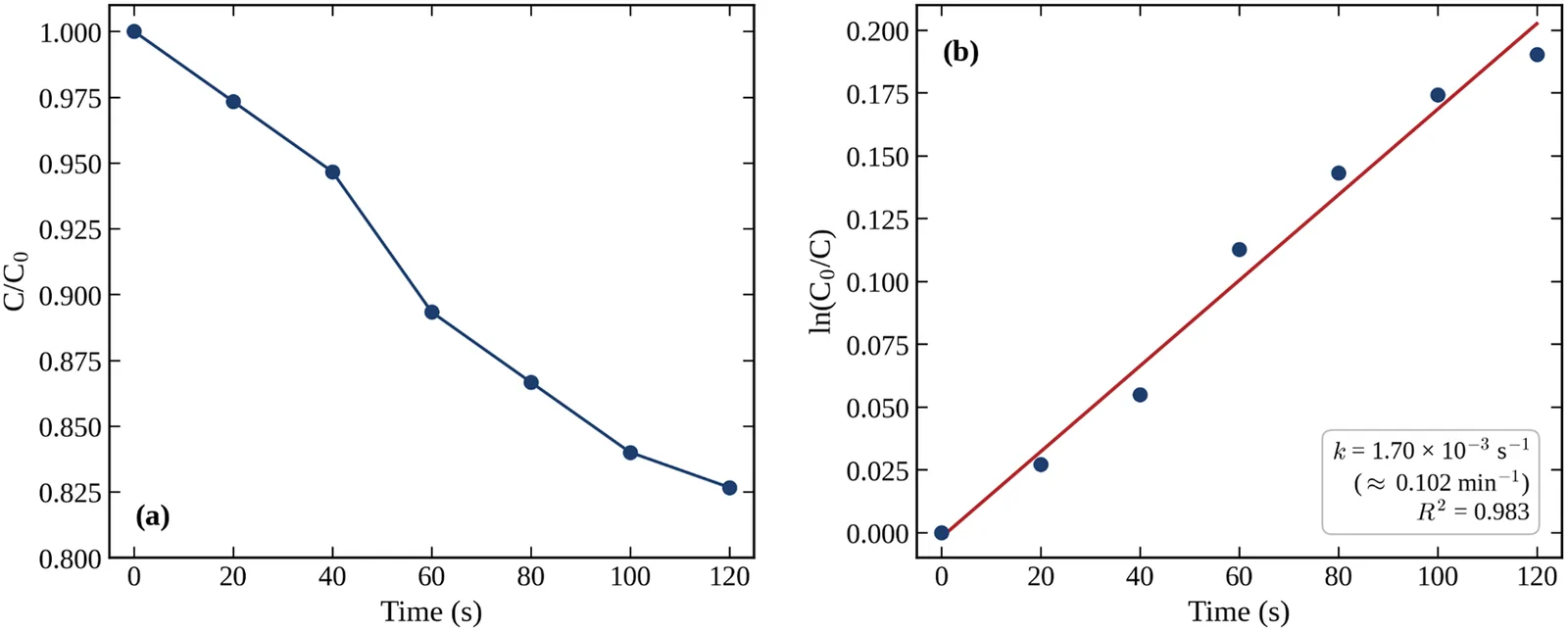
Degradation kinetics of Congo red over NiCrSe-CM in the initial reaction period: (a) *C*/*C*_0_*versus* irradiation time; (b) pseudo-first-order linear fit of ln(*C*_0_/*C*) *versus* time, giving *k* = 1.70 × 10^−3^ s^−1^ (*R*^2^ = 0.98).

The degradation kinetics of Congo red over NiCrSe-CM were evaluated in the initial low-conversion regime, where the catalyst concentration is effectively constant and the intrinsic reaction rate can be extracted without distortion from substrate depletion. The dye concentration was monitored at close 20 s intervals over the first 120 s of irradiation, corresponding to ≤∼17% conversion. As shown in Fig. 14(a), the normalised concentration (*C*/*C*_0_) decreased steadily with irradiation time.

Plotting ln(*C*_0_/*C*) against time Fig. 14(b) yielded an excellent straight line passing through the origin (intercept ≈ −0.002), confirming that the photocatalytic degradation follows pseudo-first-order kinetics in this regime. The apparent rate constant obtained from the slope was *k* = 1.70 × 10^−3^ s^−1^ (≈0.102 min^−1^) with a correlation coefficient of *R*^2^ = 0.98. Determining the rate constant from this initial linear region, rather than from the full degradation profile, provides a more reliable measure of the intrinsic photocatalytic activity of NiCrSe-CM.

#### 3.8.2 Impact of pH

**Fig. 15:**
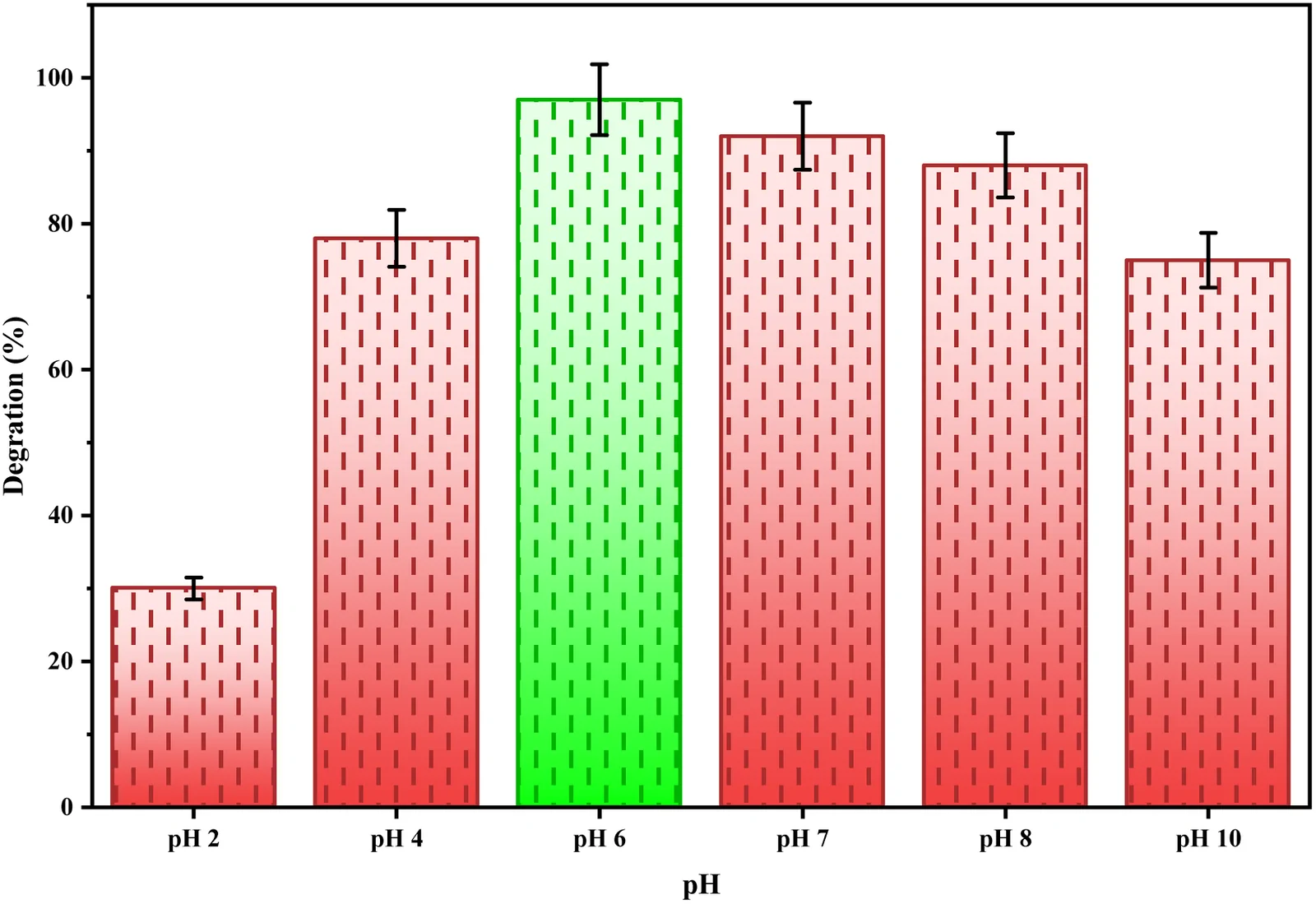
Effect of pH on the photocatalytic degradation of Congo red.

The pH affects degradation by altering the catalyst's surface charge and the ionization state of the dye. This was studied under acidic, neutral and basic conditions Fig. 15 at 60 min irradiation, 0.1 g catalyst dosage, and 20 ppm dye concentration.

Findings revealed that the maximum level of efficiency in degradation was reported at pH 6. In the acidic condition, the photocatalyst surface becomes positively charged and this increases electrostatic forces between the catalyst and anionic dye molecules. That increases the overall photocatalytic activity.^45^

On the other hand, in an alkaline environment, the anionic Congo red dye is repelled by negatively charged hydroxide ions. This repulsion reduces the interaction between the dye molecules and the catalyst, which eventually results in a reduction in the effectiveness of photocatalytic degradation.^73–75^ The degradation efficiency of CR dye at 6 pH was estimated to be 97%, making it the optimal pH level for further investigation.

**Fig. 16:**
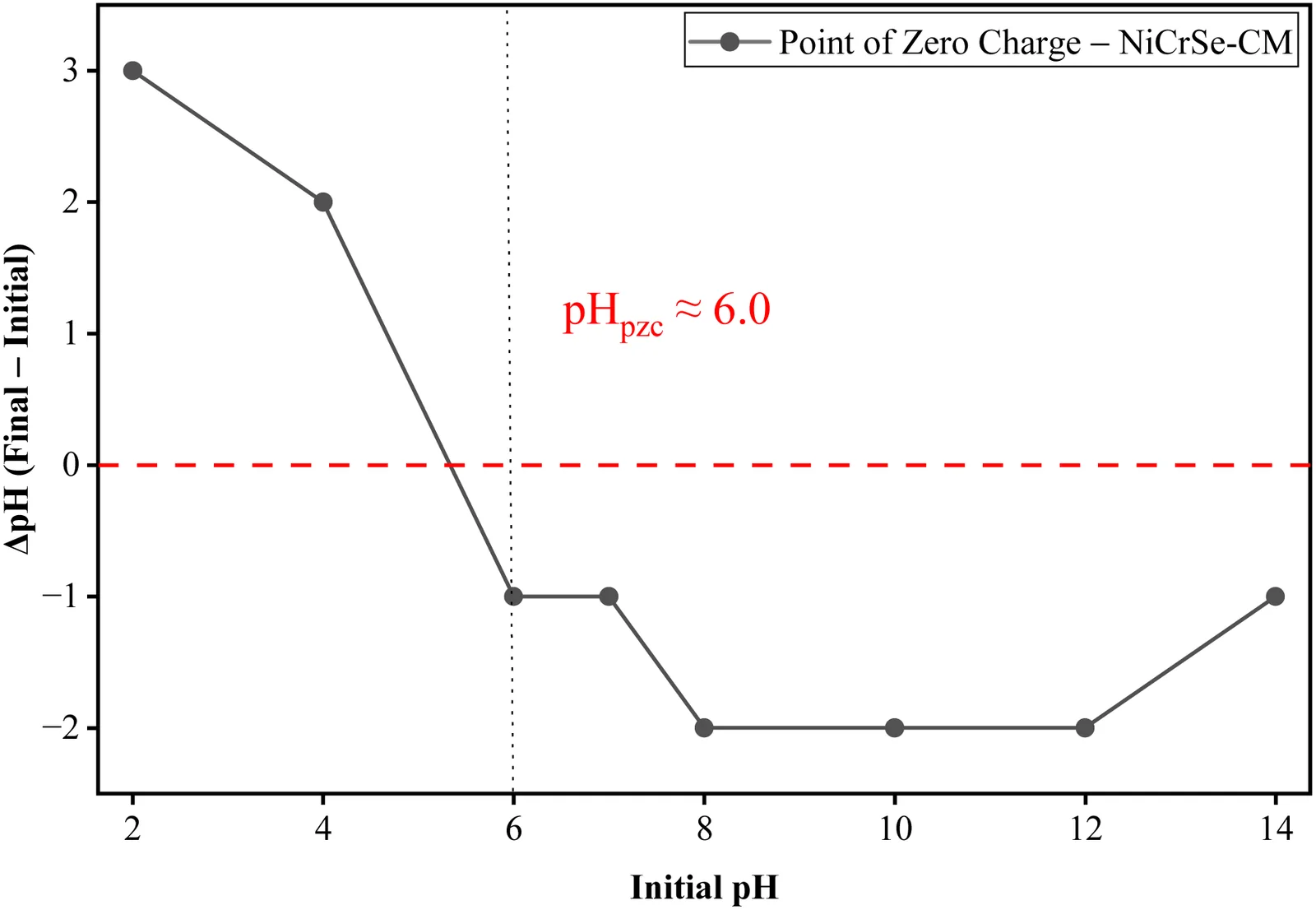
Point of zero charge (pH_pzc_) determination for NiCrSe-CM by the pH-drift method.

To further elucidate the pH-dependent behaviour, the point of zero charge (pH_pzc_) of NiCrSe-CM was determined by the pH-drift method Fig. 16.

The pH_pzc_ was found to be approximately 5.3. At solution pH values below pH_pzc_ (<5.3), the catalyst surface carries a net positive charge due to protonation of surface hydroxyl and amino groups. Since Congo red is an anionic dye carrying two sulfonate (–SO_3_^−^) groups, electrostatic attraction between the positively charged catalyst surface and the negatively charged dye molecules favours adsorption and promotes photocatalytic degradation, explaining the enhanced performance observed under acidic conditions. At pH values above pH_pzc_ (>5.3), the surface becomes negatively charged, resulting in electrostatic repulsion between the catalyst and the anionic dye, which reduces adsorption and lowers degradation efficiency. The optimum pH of 6 just above the pH_pzc_ represents a balance between sufficient surface–dye interaction and the solution chemistry of Congo red, which is most stable near neutral pH. These findings are consistent with the surface-charge mechanism reported for analogous selenide-chitosan photocatalysts.^47^

#### 3.8.3 Impact of initial dye concentrations

**Fig. 17:**
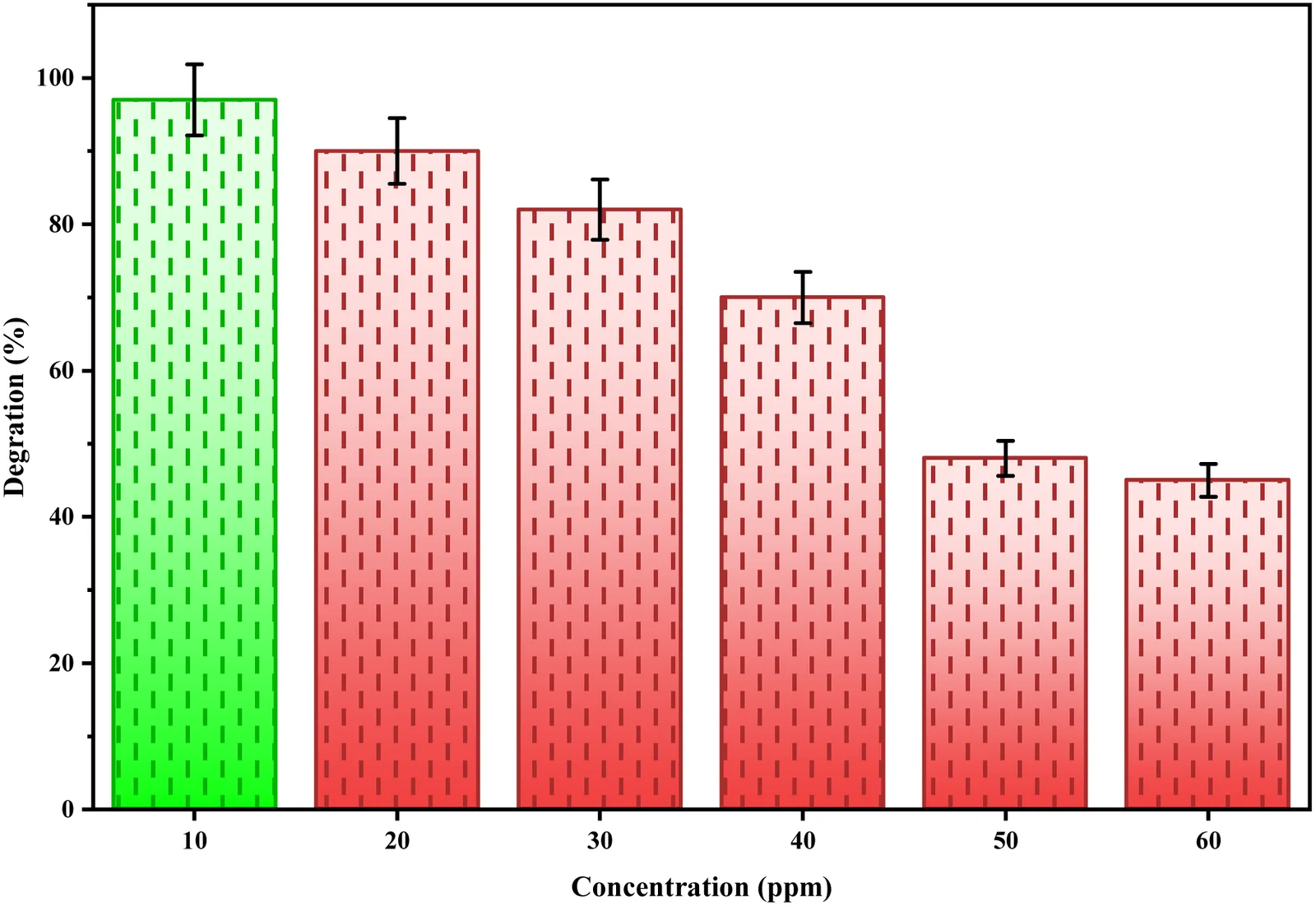
Effect of initial dye concentration on the photocatalytic degradation of Congo red.

A CR concentration range of 10–60 ppm was examined Fig. 17 at 60 min irradiation, catalyst dosage and pH 6.

At higher initial concentrations, dye molecules increasingly occupy the catalyst's active sites and absorb incident light, limiting ˙OH generation and lowering the degradation efficiency.^76–78^ The highest CR degradation (97%) occurred at 10 ppm, which was selected as the optimal concentration for subsequent experiments.

#### 3.8.4 Impact of catalyst dosage

**Fig. 18:**
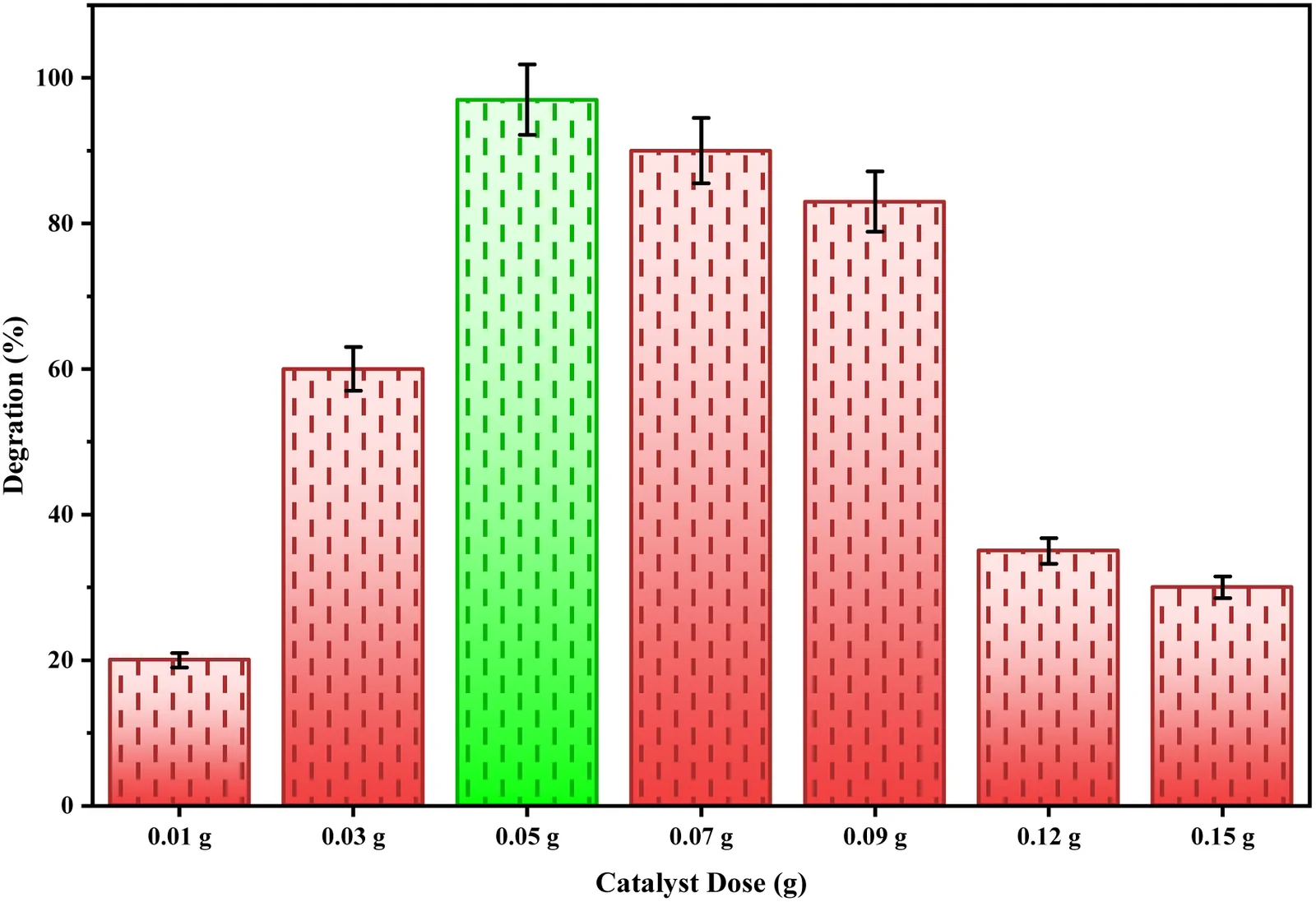
Effect of catalyst dosage on the photocatalytic degradation of Congo red.

The impact of varying NiCrSe-CM catalyst amounts (0.01–0.15 g) on CR photocatalytic degradation was studied under reaction conditions of irradiation time (60 min), CR concentration 10 ppm, and pH 6 (Fig. 18.) As shown in the results, degradation efficiency increased with the dosage of the photocatalyst with maximum 97% degradation recorded at 0.05 g (optimum). The improved adsorption and consequent photocatalytic degradation of dyes is attributed to both improvements in the amount of highly reactive hydroxyl radicals (˙OH), and augmented number of accessible surface sites. Beyond the optimum, excess catalyst causes particle aggregation and increased turbidity, which scatter and block incident light, reducing photon activation and degradation efficiency.^79^ In a heterogeneous photocatalytic system, the overall rate can be limited by mass transfer and by light (photon) transfer, and both were considered here. External mass transfer of Congo red from the bulk solution to the catalyst surface was minimised by continuous stirring, which keeps the NiCrSe-CM microspheres uniformly suspended and thins the liquid boundary layer around them. Internal mass-transfer resistance is also expected to be small, as the porous chitosan matrix and the fine dispersion of the selenide nanoparticles provide short diffusion paths and accessible active sites. With respect to light transfer, photon penetration into the suspension depends on catalyst loading: at low dosage the rate is limited by too few active sites, whereas at high dosage the suspension becomes optically dense and light scattering and shielding reduce the photon flux reaching the inner particles. This trade-off accounts for the optimum catalyst dosage observed in Fig. 18, beyond which efficiency no longer rises despite the higher catalyst amount.

#### 3.8.5 Stability and reusability study

**Fig. 19:**
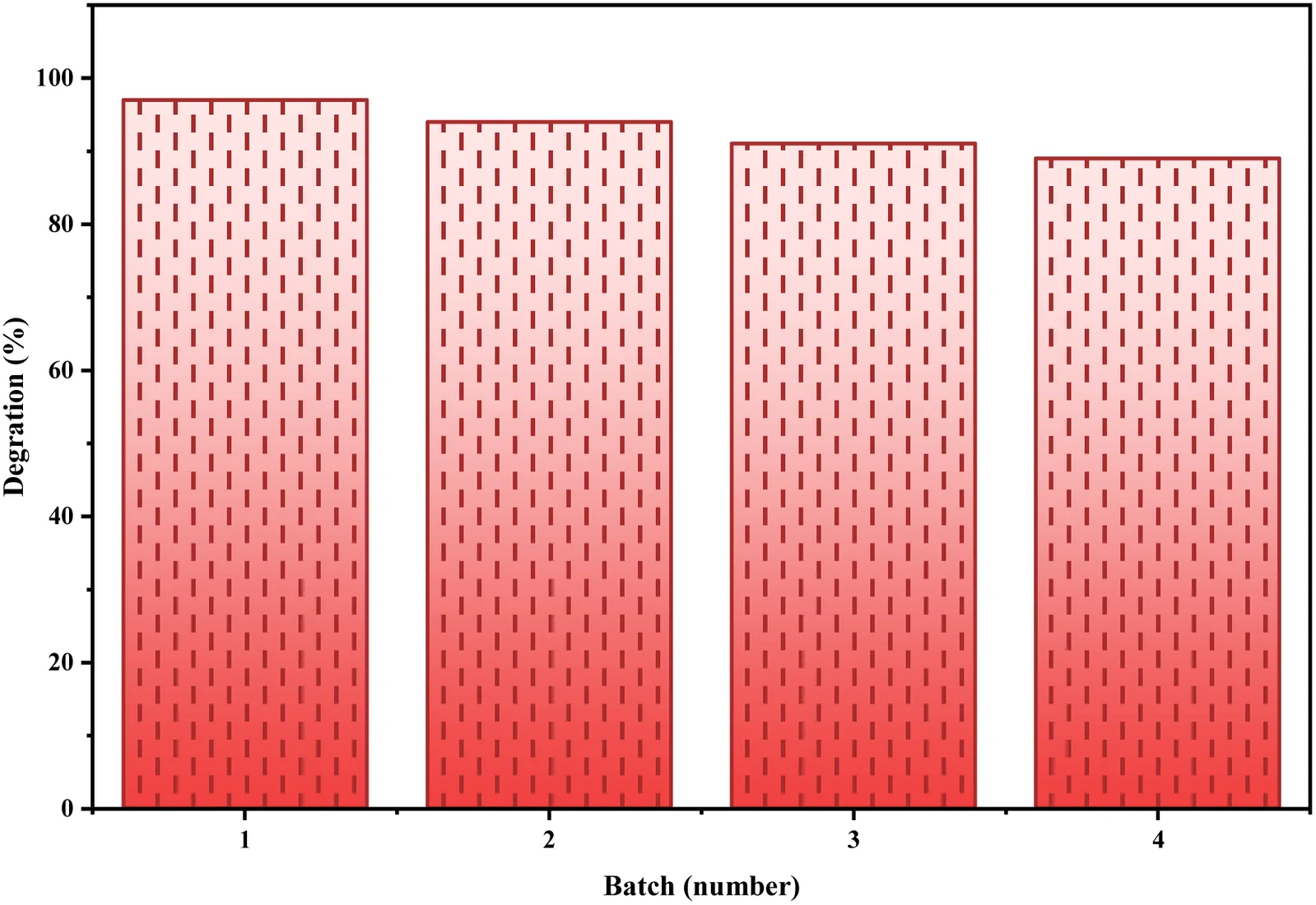
Reusability of the NiCrSe-CM catalyst over four successive cycles for Congo red degradation.

Photocatalysts should exhibit strong chemical stability, high light-harvesting efficiency, facile recovery and reuse.^80^ The Photocatalyst from the first photocatalytic cycle were washed with ethanol and distilled water, dried at 60 °C for 1.5 h, and reused under identical conditions.^73^ To assess the catalyst's capacity for recycling, NiCrSe-chitosan microspheres were reused for four successive cycles. As illustrated in Fig. 19.

The catalyst exhibited remarkable stability and efficiency over multiple runs. This gradual decrease in activity is minimal, indicating excellent photocatalytic durability and confirming the catalyst's potential for repeated application in wastewater treatment processes. Furthermore, its stability, reusability, and enhanced activity under sunlight contribute to reducing the overall cost of water treatment.^81,82^ Beyond its photocatalytic performance, the process is also cost-effective and environmentally benign. Because the selenide nanoparticles are immobilised within chitosan microspheres, the catalyst can be recovered by simple filtration and reused over 4 successive cycles with only a small loss of activity (Fig. 19), lowering the effective cost per treatment cycle and avoiding the difficult separation and potential release of free nanoparticles. The use of inexpensive precursors, a simple co-precipitation synthesis in aqueous medium, and natural sunlight instead of energy-intensive UV lamps further reduce the operating cost, while chitosan itself is a biodegradable, non-toxic and naturally abundant biopolymer.

#### 3.8.6 Post-catalytic FTIR

**Fig. 20:**
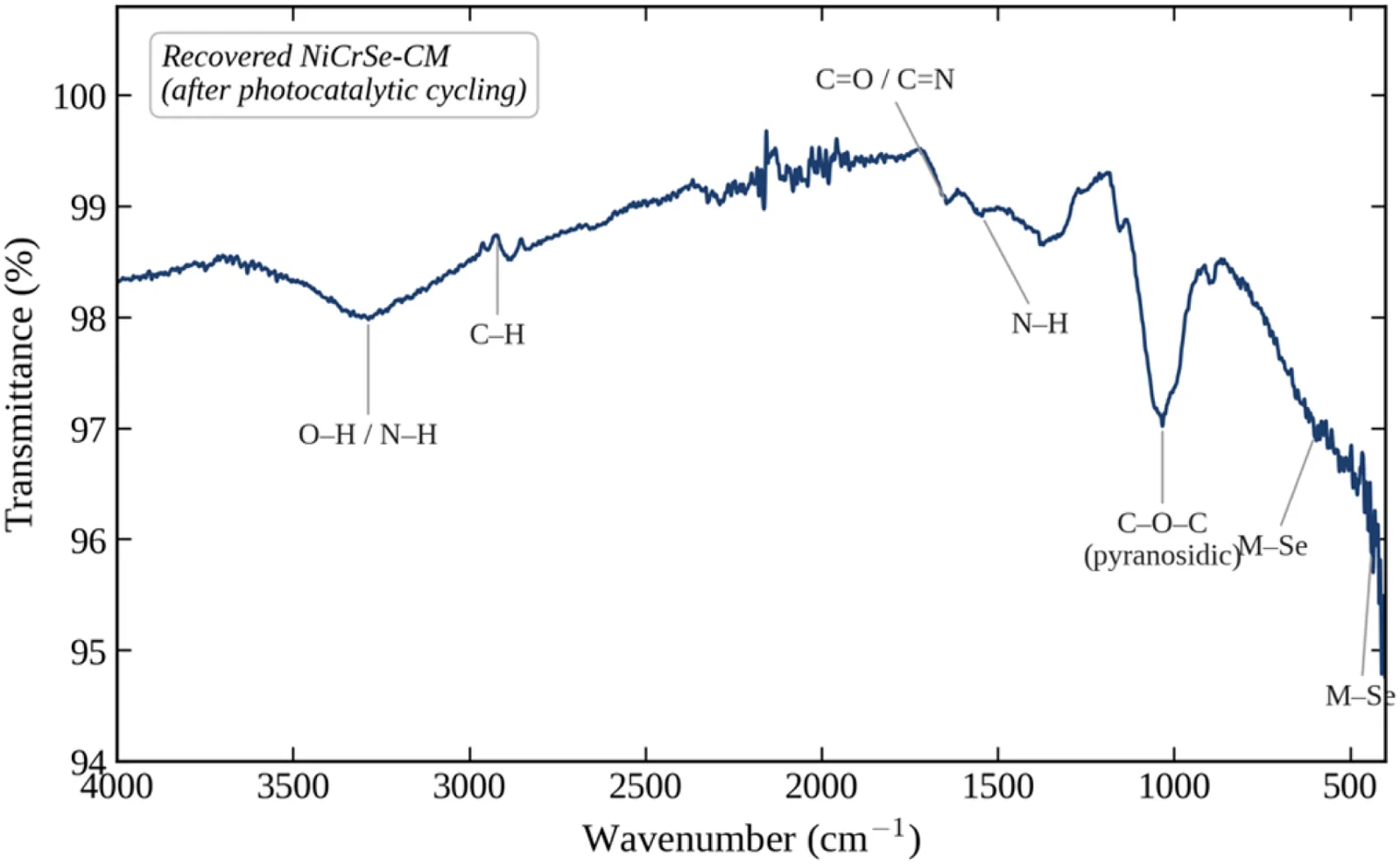
Post-catalytic FTIR spectrum of the NiCrSe-CM photocatalyst recovered.

To assess the structural and chemical stability of the photocatalyst after repeated use, the FTIR spectrum of the NiCrSe-CM microspheres recovered after the reusability cycles was recorded (Fig. 20) and compared with that of the fresh composite (Fig. 2).

The spent catalyst retained all of the characteristic vibrational features of the as-prepared material. The broad band centred at ∼3287 cm^−1^ (O–H/N–H stretching), the weak C–H stretching features near 2920 cm^−1^, and the bands at ∼1645 cm^−1^ (overlapping C=O/C=N stretching of the amide and Schiff-base linkages) and ∼1546 cm^−1^ (N–H bending) confirm that the chitosan backbone and the cross-linked microsphere framework remained intact. The pyranosidic-ring and glycosidic C–O–C vibrations of chitosan were preserved in the 1080–1033 cm^−1^ region, while the metal–selenium (M–Se) stretching vibrations characteristic of the ternary selenide were still clearly observed below ∼650 cm^−1^ (≈600 and ≈440 cm^−1^). The minor shifts in band position (*e.g.*, 3361 → 3287 and 630 → 600 cm^−1^) and small intensity changes are attributed to surface interactions with residual adsorbed dye fragments and reorganisation of surface hydroxyl/amine groups rather than to decomposition of the composite. The retention of both the chitosan-derived and M–Se bands demonstrates that NiCrSe-CM preserves its chemical structure and composite integrity after photocatalytic operation, consistent with the high degradation efficiency maintained over successive cycles and confirming its stability and reusability.

Post-cycle XRD analysis could not be performed owing to the temporary unavailability of X-ray diffraction facilities; however, the retention of the characteristic FTIR bands, together with the sustained reusability performance, confirms the structural stability of the catalyst.

#### 3.8.7 Scavenger/active-species results

**Fig. 21:**
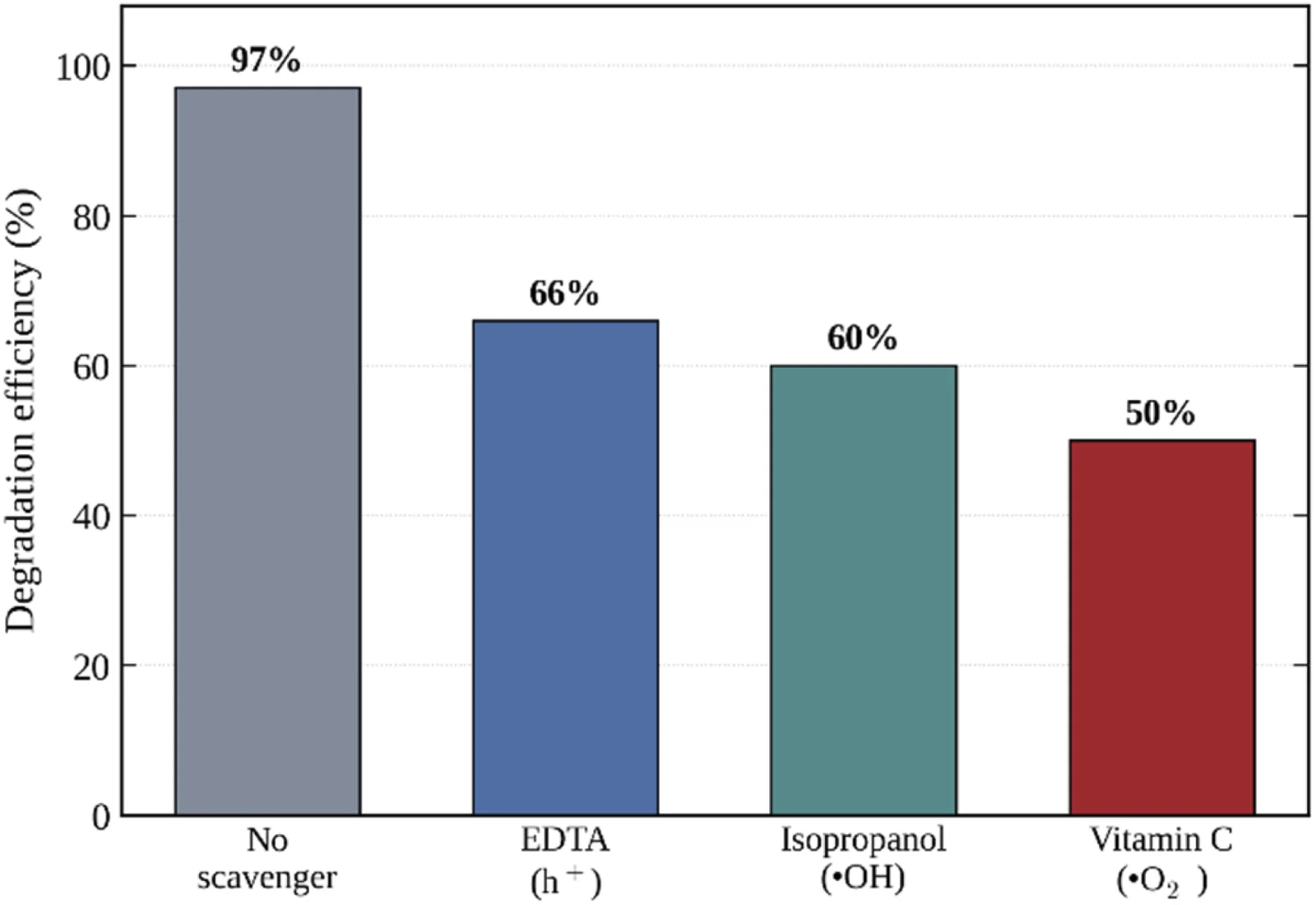
Effect of different radical scavengers (EDTA for h^+^, isopropanol for ˙OH, ascorbic acid for ˙O_2_^−^) on the photocatalytic degradation of Congo red over NiCrSe-CM under optimum conditions.

To identify the principal reactive species responsible for Congo red degradation, radical-trapping (scavenger) experiments were carried out under the optimum conditions using ethylenediaminetetraacetic acid (EDTA) as a hole (h^+^) scavenger, isopropanol (IPA) as a hydroxyl-radical (˙OH) scavenger, and ascorbic acid (vitamin C) as a superoxide-radical (˙O_2_^−^) scavenger Fig. 21.

In the absence of any scavenger, 97% degradation was achieved. The efficiency decreased to 66% on addition of EDTA, to 60% with isopropanol, and to 50% with ascorbic acid, corresponding to suppressions of 31, 37 and 47 percentage points, respectively. The most pronounced inhibition occurred with ascorbic acid, indicating that superoxide radicals (˙O_2_^−^) are the dominant reactive species governing the degradation, followed by hydroxyl radicals (˙OH) and photogenerated holes (h^+^). The appreciable suppression caused by all three scavengers confirms that ˙O_2_^−^, ˙OH and h^+^ each contribute to the process, with superoxide playing the leading role. This is fully consistent with the band-edge positions obtained from the Mott–Schottky analysis: the conduction-band edge of NiCrSe-CM (−0.93 V *vs.* NHE) is sufficiently negative to reduce dissolved O_2_ to ˙O_2_^−^, while the valence-band holes contribute through direct oxidation and the formation of secondary ˙OH.

#### 3.8.8 Proposed photocatalytic mechanism

The proposed photocatalytic degradation mechanism is based on the band-edge positions determined from the Mott–Schottky analysis (Section 3.6). For NiCrSe-CM, the conduction- and valence-band edges lie at *E*_β_ ≈ −0.93 V and *E*_vβ_ ≈ +1.27 V *vs.* NHE, respectively, consistent with the established framework for locating semiconductor band edges relative to solution redox couples.^83,84^ Under solar irradiation, NiCrSe-CM absorbs photons of energy equal to or greater than its band gap and is excited to generate electron–hole pairs (eqn (10)). Because the conduction-band edge (−0.93 V) is more negative than the O_2_/˙O_2_^−^ redox couple (−0.33 V *vs.* NHE), the photogenerated conduction-band electrons reduce dissolved oxygen to superoxide radicals (˙O_2_^−^) (eqn. (11)), which are subsequently protonated and converted through ˙OOH and H_2_O_2_ into hydroxyl radicals (˙OH) (eqn (12)–(14)). In contrast, because the valence-band edge (+1.27 V) is less positive than the ˙OH/OH^−^ couple (+1.99 V *vs.* NHE), the valence-band holes cannot oxidise water or hydroxide to ˙OH directly; instead, they act as direct oxidants towards the adsorbed Congo red molecules (eqn (15)). The combined action of ˙O_2_^−^, photogenerated holes (h^+^) and secondary ˙OH attacks the azo (–N=N–) linkages and aromatic rings of Congo red, leading to progressive fragmentation and partial mineralisation into CO_2_, H_2_O and inorganic ions (eqn (16)). This pathway is consistent with the radical-trapping (scavenger) experiments, which identified superoxide radicals (˙O_2_^−^) as the principal reactive species governing Congo red degradation.^47^

10 NiCrSe-CM + *hν* → e_⊂β_^−^ + h_vβ_^+^

11 e_⊂β_^−^ + O_2_ → ˙O_2_^−^

12 ˙O_2_^−^ + H^+^ → ˙OOH

13 2˙OOH → H_2_O_2_ + O_2_

14 H_2_O_2_ + e_⊂β_^−^ → ˙OH + OH^−^

15 h_vβ_^+^ + Congo red → oxidation products

16 (˙O_2_^−^ + ˙OH + h^+^) + Congo red → intermediates → CO_2_ + H_2_O + NO_3_^−^/SO_4_^2−^

### 3.9 Statistical analysis and modeling

**Fig. 22:**
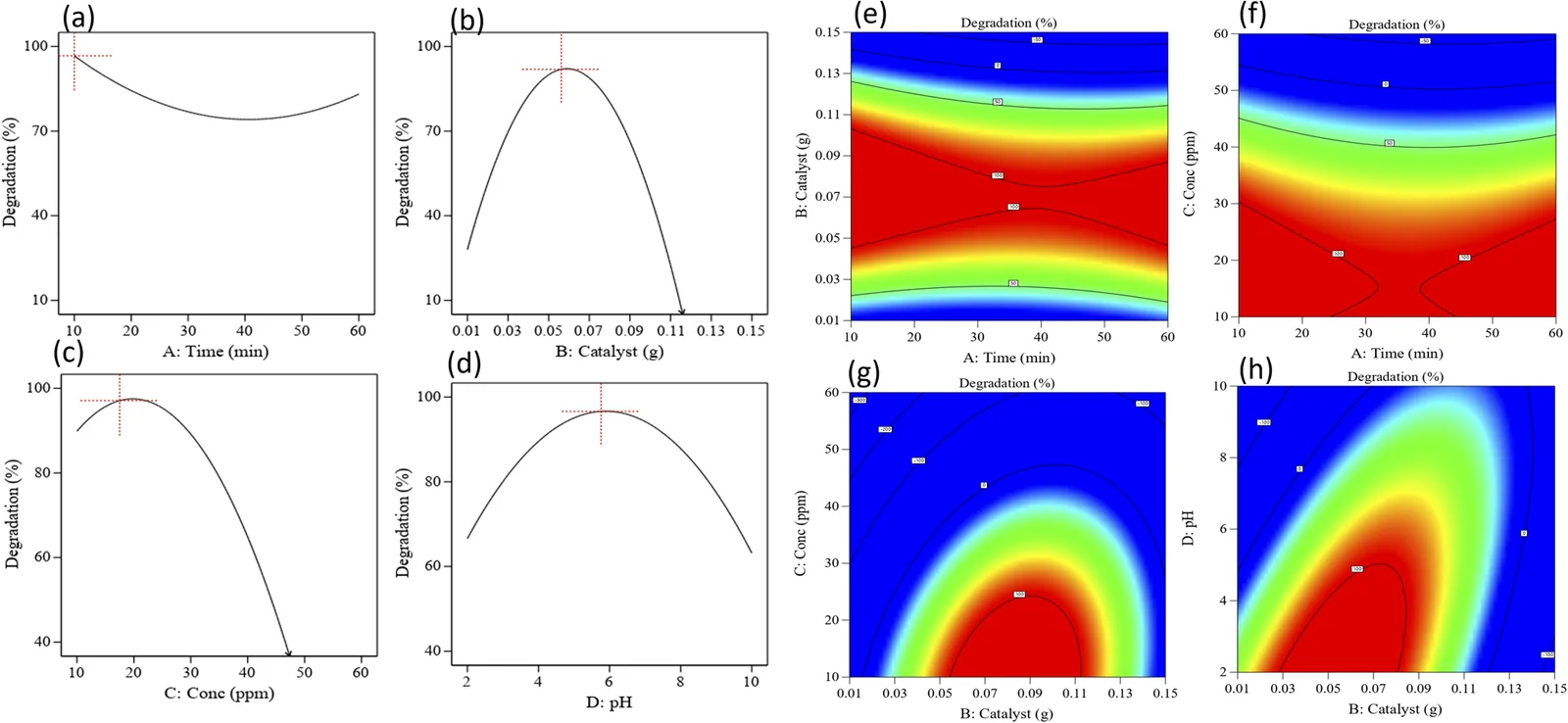
One-factor-at-a-time (OFAT) plots showing the individual effects of (a) irradiation time, (b) catalyst dosage, (c) dye concentration and (d) pH on Congo red degradation, and (e–h) two-dimensional contour plots showing the combined effects of these parameters, using NiCrSe-CM.

The effect of operational parameters on the photocatalytic degradation of CR dye was evaluated using RSM, and the developed quadratic regression model (eqn (6)) describes the relationship between process variables and degradation efficiency. The software used for RSM was DesignXpert V13. The model indicates that both individual variables and their interaction terms significantly influence CR degradation, with catalyst dosage exhibiting the most pronounced positive effect, highlighting its dominant role in enhancing photocatalytic performance. Fig. 22 illustrates the influence of individual parameters and their interactions on CR degradation efficiency.

The OFAT plots (Fig. 22a–d) demonstrate the individual effects of irradiation time, catalyst dosage, dye concentration, and pH on degradation efficiency. Maximum degradation was observed at 0.05 g catalyst dosage, 10 ppm dye concentration, 60 min irradiation time, and pH 6, indicating the optimum operating conditions. Furthermore, the contour plots (Fig. 22e–h) illustrate the combined influence of parameters, where maximum degradation efficiency is achieved within specific ranges, particularly at catalyst dosage between 0.05–0.11 g and irradiation time between 10–60 min, as well as dye concentration between 10–30 ppm.

**Fig. 23:**
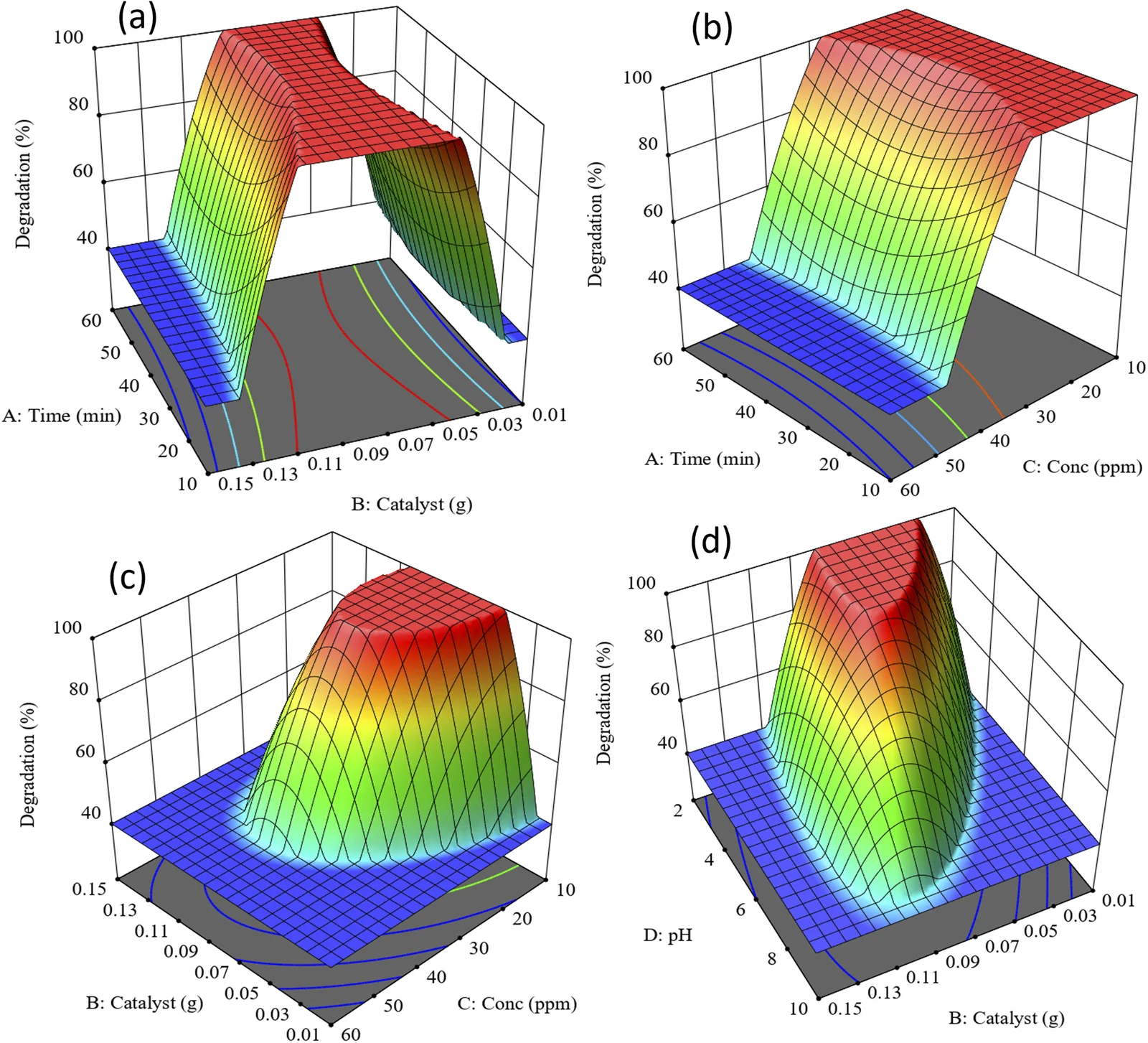
(a–d) Three-dimensional response-surface plots of Congo red degradation using NiCrSe-CM.

Fig. 23 presents the three-dimensional response surface plots illustrating the interaction between process variables. The 3D response surfaces demonstrate that maximum CR degradation is achieved when all parameters are maintained within their optimal ranges. The curvature of the surfaces indicates strong nonlinear interactions between variables, confirming the suitability of the quadratic model in describing the system behaviour.

The statistical significance and adequacy of the developed model were further evaluated using ANOVA, as presented in Table 3.

**Table 3:** Analysis of variance (ANOVA) for the quadratic model of Congo red degradation efficiency

Source	Sum of squares	DF	Mean square	*F*-Value	*p*-Value	Significance
Model	13717.15	14	979.80	106.33	<0.0001	Significant
*A*-time	79.89	1	79.89	8.67	0.0100	
*B*-catalyst	0.1233	1	0.1233	0.0134	0.9095	
*C*-conc.	62.36	1	62.36	6.77	0.0200	
*D*-pH	24.19	1	24.19	2.62	0.1260	
AB	34.92	1	34.92	3.79	0.0706	
AC	51.99	1	51.99	5.64	0.0313	
AD	84.79	1	84.79	9.20	0.0084	
BC	19.34	1	19.34	2.10	0.1680	
BD	41.47	1	41.47	4.50	0.0509	
CD	429.10	1	429.10	46.57	<0.0001	
*A* ^2^	407.68	1	407.68	44.24	<0.0001	
*B* ^2^	207.85	1	207.85	22.56	0.0003	
*C* ^2^	451.47	1	451.47	49.00	<0.0001	
*D* ^2^	1072.03	1	1072.03	116.34	<0.0001	
Residual	138.22	15	9.21			
Lack of fit	77.72	5	15.54	2.57	0.0958	Not significant
Pure error	60.50	10	6.05			
Cor. total	13855.37	29				

The ANOVA results reveal a high *F*-value of 106.33, indicating that the model is highly significant with a very low probability of error (<0.0001). Most of the model terms satisfy the significance criterion (*p* < 0.05), confirming their substantial contribution to CR degradation. Additionally, the non-significant lack-of-fit value demonstrates that the model fits the experimental data well. The central composite design was used to optimize the parameters across the experimental runs shown in Table 4, while the fitting and predictive capability of the model are summarized in Table 5.

**Table 4:** Central composite design RSM runs with process variables and % degradation response

Std.	Run	*A*: time min	*B*: catalyst g	*C*: conc. ppm	*D*: pH	Degradation %
4	1	10	0.05	10	6	97
21	2	20	0.05	10	10	75
18	3	30	0.05	10	6	80
24	4	40	0.05	20	6	86
17	5	50	0.05	10	6	92
9	6	60	0.05	10	6	97
2	7	10	0.01	10	2	20
28	8	60	0.03	30	6	60
27	9	60	0.05	10	6	97
8	10	40	0.07	10	6	90
30	11	60	0.09	10	6	83
10	12	10	0.12	10	6	45
14	13	60	0.05	10	6	97
29	14	60	0.05	10	6	97
15	15	30	0.05	20	7	88
6	16	60	0.05	10	6	97
23	17	60	0.05	40	6	70
5	18	10	0.05	50	6	48
11	19	60	0.05	60	4	45
13	20	60	0.05	10	2	30
1	21	60	0.05	10	6	97
16	22	40	0.05	20	6	97
7	23	60	0.05	10	6	97
20	24	60	0.05	10	6	97
12	25	40	0.05	10	10	75
22	26	60	0.05	10	6	97
25	27	20	0.05	10	4	75
26	28	40	0.05	10	6	80
19	29	30	0.05	10	7	86
3	30	50	0.05	10	6	92

**Table 5:** Fit statistics of the quadratic regression model for Congo red degradation efficiency

Std. dev.	3.04	*R* ^2^	0.9900
Mean	79.57	Adjusted *R*^2^	0.9807
C.V. %	3.82	Predicted *R*^2^	0.971
		Adeq precision	36.4684

The high coefficient of determination (*R*^2^ = 0.9900), along with strong adjusted and predicted *R*^2^ values, indicates excellent agreement between experimental and predicted results. Moreover, the low coefficient of variation (3.82%) and high adequate precision value (36.4684) confirm the reliability and robustness of the developed model.

**Fig. 24:**
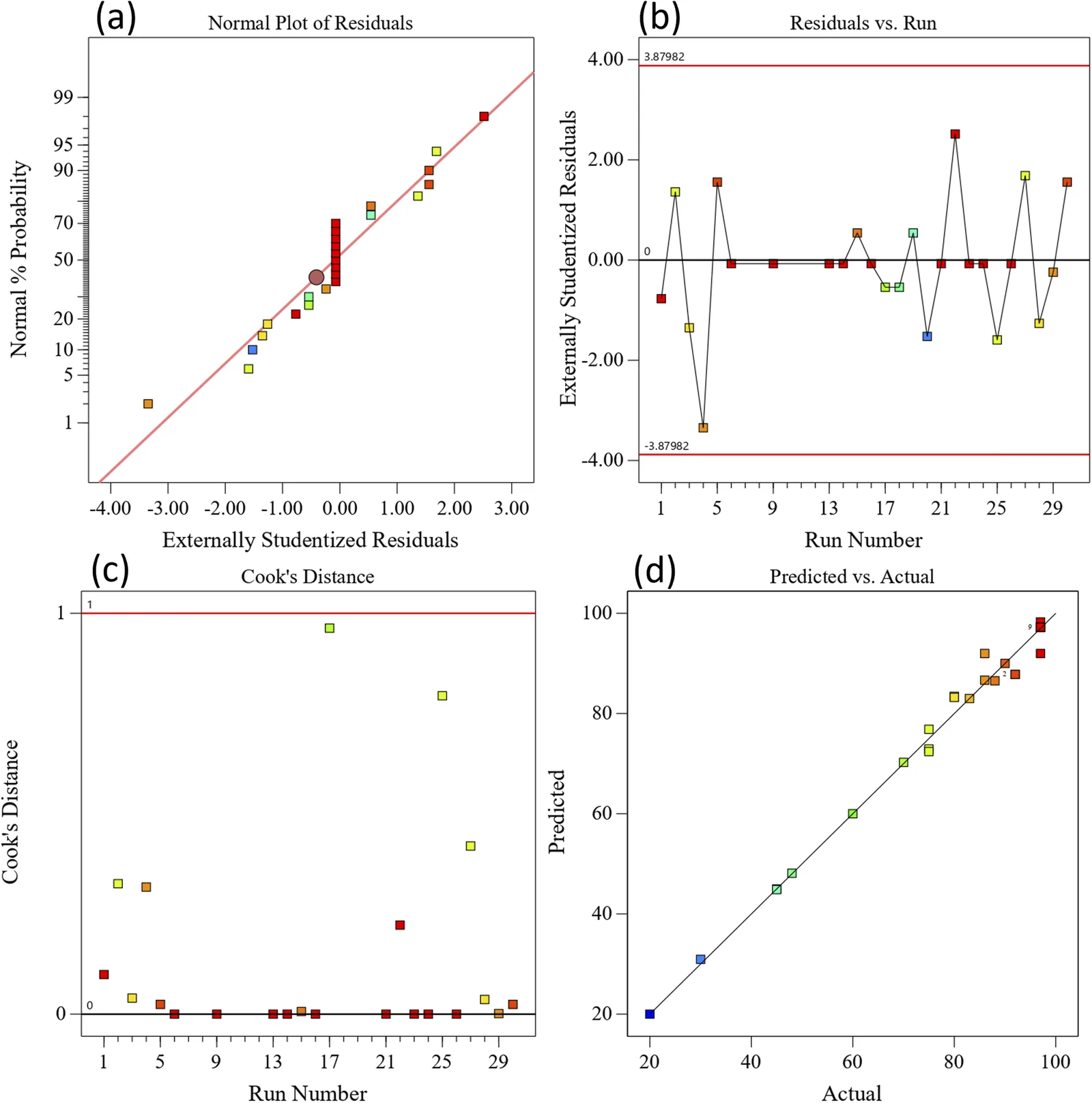
(a–d) Diagnostic plots (normal probability of residuals, residuals *vs.* predicted, and predicted *vs.* actual) for the Congo red degradation model.

Fig. 24 presents the diagnostic plots used to assess model adequacy. The diagnostic plots demonstrate that the residuals are normally distributed and fall within acceptable limits, indicating the absence of significant outliers. Furthermore, the actual *versus* predicted plot shows strong agreement, confirming the accuracy and validity of the proposed model in predicting CR degradation efficiency.

## 4 Conclusions

In this work, ternary metal selenide-chitosan microspheres were successfully synthesized and demonstrated as an efficient photocatalyst for the degradation of Congo red (CR) dye under solar irradiation. The prepared nanomaterial exhibited a suitable optical band gap of 2.2 eV, confirming its strong absorption in the visible (green) region and suitability for solar-driven applications. The photocatalytic studies revealed that maximum degradation efficiency of approximately 97% was achieved under optimized conditions of 0.05 g catalyst dosage, 10 ppm initial dye concentration, pH 6, and 60 min irradiation time. The RSM-based optimization confirmed that catalyst dosage plays a dominant role, while strong synergistic interactions among process variables significantly influence the overall performance. The developed quadratic model demonstrated excellent statistical reliability with *R*^2^ = 0.9900, supported by low variability (CV = 3.82%) and high signal-to-noise ratio (Adeq Precision = 36.4684). Furthermore, ANN and SVM models showed strong agreement with experimental results, validating the robustness and predictive capability of the system. Overall, the combination of a suitable band gap, high degradation efficiency, and strong model validation highlights the potential of the synthesized photocatalyst as a promising candidate for efficient and sustainable wastewater treatment applications.

## Author contributions

Nawaz Khan: conceptualization, methodology, investigation and original draft. Waqar Ahmad: visualization, data curation, writing – original draft. Muhammad Khan: editing, writing, review. Adnan Khan: (supervision, conceptualization writing – original draft, visualization). Mahnoor Amjad: statistical analysis and modelling. Sabir Khan: investigate and supervised specific finding of the work. Nauman Ali: verified analytical methods. Nisar Ali: writing – review and editing. Jiaojiao Zheng: supervision, methodology, review and editing.

## Conflicts of interest

The authors declare no conflicts of interest.

## Supplementary Material

RA-016-D6RA04309G-s001

## Data Availability

The data supporting this study are available within the article and its supplementary information (SI) file. Additional data related to this work are available from the corresponding author upon reasonable request. Supplementary information is available. See DOI: https://doi.org/10.1039/d6ra04309g.

## References

[cit1] Li H. (2022). *et al.*, Synthesis of bimetallic FeCu-MOF and its performance as catalyst of peroxymonosulfate for degradation of methylene blue. Materials.

[cit2] Tanaka K., Padermpole K., Hisanaga T. (2000). Photocatalytic degradation of commercial azo dyes. Water Res..

[cit3] Sarkar S. (2020). *et al.*, Green polymeric nanomaterials for the photocatalytic degradation of dyes: a review. Environ. Chem. Lett..

[cit4] Ahmadijokani F. (2024). *et al.*, COF and MOF hybrids: advanced materials for wastewater treatment. Adv. Funct. Mater..

[cit5] Mukherjee D., Van der Bruggen B., Mandal B. (2022). Advancements in visible light responsive MOF composites for photocatalytic decontamination of textile wastewater: A review. Chemosphere.

[cit6] Wu Z. (2017). *et al.*, Photocatalytic decontamination of wastewater containing organic dyes by metal–organic frameworks and their derivatives. ChemCatChem.

[cit7] Rafiq A. (2021). *et al.*, Photocatalytic degradation of dyes using semiconductor photocatalysts to clean industrial water pollution. J. Ind. Eng. Chem..

[cit8] Kansal S. K., Kaur N., Singh S. (2009). Photocatalytic degradation of two commercial reactive dyes in aqueous phase using nanophotocatalysts. Nanoscale Res. Lett..

[cit9] Shahriyari Far H. (2023). *et al.*, Synthesis of MXene/Metal-Organic Framework (MXOF) composite as an efficient photocatalyst for dye contaminant degradation. Inorg. Chem. Commun..

[cit10] El-Sayed F. (2022). *et al.*, Comparative Degradation Studies of Carmine Dye by Photocatalysis and Photoelectrochemical Oxidation Processes in the Presence of Graphene/N-Doped ZnO Nanostructures. Crystals.

[cit11] Khan M. (2021). *et al.*, Development and characterization of regenerable chitosan-coated nickel selenide nano-photocatalytic system for decontamination of toxic azo dyes. Int. J. Biol. Macromol..

[cit12] Xie K. (2022). *et al.*, Progress of graphite carbon nitride with different dimensions in the photocatalytic degradation of dyes: A review. J. Alloys Compd..

[cit13] Aljuaid A. (2023). *et al.*, g-C3N4 Based Photocatalyst for the Efficient Photodegradation of Toxic Methyl Orange Dye: Recent Modifications and Future Perspectives. Molecules.

[cit14] Kaur H. (2023). *et al.*, Metal–organic framework-based materials for wastewater treatment: superior adsorbent materials for the removal of hazardous pollutants. ACS Omega.

[cit15] Siddiqui S. I. (2023). *et al.*, Investigation of Congo red toxicity towards different living organisms: a review. Processes.

[cit16] Oladoye P. O. (2022). *et al.*, Toxicity and decontamination strategies of Congo red dye. Groundw. Sustain. Dev..

[cit17] El-Meanawy A., Mueller C., Iczkowski K. A. (2019). Improving sensitivity of amyloid detection by Congo red stain by using polarizing microscope and avoiding pitfalls. Diagn. Pathol..

[cit18] Harja M., Buema G., Bucur D. (2022). Recent advances in removal of Congo Red dye by adsorption using an industrial waste. Sci. Rep..

[cit19] Saratale R. G. (2011). *et al.*, Bacterial decolorization and degradation of azo dyes: A review. J. Taiwan Inst. Chem. Eng..

[cit20] Chen H. (2006). Recent advances in azo dye degrading enzyme research. Curr. Protein Pept. Sci..

[cit21] DeVito S. C. (2018). The Need for, and the Role of the Toxicological Chemist in the Design of Safer Chemicals. Toxicol. Sci..

[cit22] Mondol M. , Rahman M. M. S. and Angon P. B., Removal of Congo Red Dye by Using Chemically Activated Lemon Peel Adsorbent, 2024, preprint, 10.21203/rs.3.rs-4720412/v1.

[cit23] Lafi R., Montasser I., Hafiane A. (2019). Adsorption of congo red dye from aqueous solutions by prepared activated carbon with oxygen-containing functional groups and its regeneration. Adsorpt. Sci. Technol..

[cit24] Barani A., Alizadeh S. R., Ebrahimzadeh M. A. (2023). A comprehensive review on catalytic activities of green-synthesized selenium nanoparticles on dye removal for wastewater treatment. Water.

[cit25] Manzoor M. H. (2024). *et al.*, Wastewater treatment using Metal-Organic Frameworks (MOFs). Appl. Mater. Today.

[cit26] Langa C. D., Mabuba N., Hintsho-Mbita N. C. (2025). Current progress in the biosynthesis of metal sulfide nanomaterials for the degradation of dyes: a review. Catalysts.

[cit27] Aina A. R. N. (2025). *et al.*, Recent advances in ZnO based photocatalysts for industrial dye degradation. Discover Appl. Sci..

[cit28] Oveisi M., Alinia Asli M., Mahmoodi N. M. (2019). Carbon nanotube based metal-organic framework nanocomposites: Synthesis and their photocatalytic activity for decolorization of colored wastewater. Inorg. Chim. Acta.

[cit29] Emmanuel S. S. (2024). *et al.*, A Pragmatic Review on Bio-polymerized Metallic Nano-Architecture for Photocatalytic Degradation of Recalcitrant Dye Pollutants. J. Polym. Environ..

[cit30] Smrithi S. P., Kottam N., Vergis B. R. (2025). Heteroatom Modified Hybrid Carbon Quantum Dots Derived from Cucurbita pepo for the Visible Light Driven Photocatalytic Dye Degradation. Top. Catal..

[cit31] Pascariu P. (2019). *et al.*, Novel rare earth (RE-La, Er, Sm) metal doped ZnO photocatalysts for degradation of Congo-Red dye: Synthesis, characterization and kinetic studies. J. Environ. Manage..

[cit32] Rauf M. A., Ashraf S. S. (2009). Fundamental principles and application of heterogeneous photocatalytic degradation of dyes in solution. Chem. Eng. J..

[cit33] Sahoo C., Gupta A. K., Pal A. (2005). Photocatalytic degradation of Crystal Violet (C.I. Basic Violet 3) on silver ion doped TiO2. Dyes Pigm..

[cit34] Peerakiatkhajohn P. (2021). *et al.*, Efficient and Rapid Photocatalytic Degradation of Methyl Orange Dye Using Al/ZnO Nanoparticles. Nanomaterials.

[cit35] Khosravi-Far M. (2025). *et al.*, ZIF-8/Bi2O3/Ag6Si2O7 nanocomposite as a novel sono-photocatalyst for the efficient degradation of malachite green. Colloids Surf., A.

[cit36] Taghavi S. M. (2026). *et al.*, Photocatalytic nanocomposites: Mechanisms and influencing parameters to practical challenges. Chem. Eng. J..

[cit37] Issahaku I., Tetteh I. K., Tetteh A. Y. (2023). Chitosan and chitosan derivatives: Recent advancements in production and applications in environmental remediation. Environ. Adv..

[cit38] Goci M. C. (2023). *et al.*, Chitosan-Based Polymer Nanocomposites for Environmental Remediation of Mercury Pollution. Polymers.

[cit39] Jiang R. (2024). *et al.*, A review on chitosan/metal oxide nanocomposites for applications in environmental remediation. Int. J. Biol. Macromol..

[cit40] Gadore V., Mishra S. R., Ahmaruzzaman M. (2024). Recent developments in transition metal selenide-based materials for energy storage, conversion, generation, environmental remediation, and sensing. J. Inorg. Organomet. Polym. Mater..

[cit41] Nawaz A. (2023). *et al.*, Solar light driven degradation of textile dye contaminants for wastewater treatment–studies of novel polycationic selenide photocatalyst and process optimization by response surface methodology desirability factor. Chemosphere.

[cit42] Bharucha S. R. (2025). *et al.*, Sunlight driven NbSe2 photocatalyst for efficient degradation of Janus green and methylene blue dyes. Sci. Rep..

[cit43] Yang Y. (2021). *et al.*, Insights into the mechanism of enhanced photocatalytic dye degradation and antibacterial activity over ternary ZnO/ZnSe/MoSe2 photocatalysts under visible light irradiation. Appl. Surf. Sci..

[cit44] Jahan F. (2025). *et al.*, Novel Z-scheme 2D/3D photocatalyst: Bismuth vanadate/zinc selenide for enhanced photocatalytic degradation of organic pollutants. Surf. Interfaces.

[cit45] Azam J. (2024). *et al.*, Solar Light Assisted Metal Selenide-Chitosan Nanoparticles from Structure Tailoring to Photocatalytic Mineralization of Selected Industrial Dyes. Water, Air, Soil Pollut..

[cit46] Khan S. (2021). *et al.*, Degradation of Congo red dye using ternary metal selenide-chitosan microspheres as robust and reusable catalysts. Environ. Technol. Innovation.

[cit47] Yang Y. (2021). *et al.*, Chitosan-capped ternary metal selenide nanocatalysts for efficient degradation of Congo red dye in sunlight irradiation. Int. J. Biol. Macromol..

[cit48] Das S. (2025). *et al.*, Artificial neural network modeling of photocatalytic degradation of pollutants: a review of photocatalyst, optimum parameters and model topology. Catal. Rev..

[cit49] Anandhi G., Iyapparaja M. (2024). Photocatalytic degradation of drugs and dyes using a machine learning approach. RSC Adv..

[cit50] Qirui L. (2025). *et al.*, Ternary metal oxide–chitosan hybrids for efficient photocatalytic remediation of organic pollutants from wastewater. Top. Catal..

[cit51] Ayaz A. (2022). *et al.*, Comparison of ground-based global horizontal irradiance and direct normal irradiance with satellite-based suny model. Energies.

[cit52] Nawaz A. (2020). *et al.*, Fabrication and characterization of new ternary ferrites-chitosan nanocomposite for solar-light driven photocatalytic degradation of a model textile dye. Environ. Technol. Innovation.

[cit53] Yang Y. (2021). *et al.*, Chitosan-capped ternary metal selenide nanocatalysts for efficient degradation of Congo red dye in sunlight irradiation. Int. J. Biol. Macromol..

[cit54] Berkani M. (2020). *et al.*, Photocatalytic degradation of industrial dye in semi-pilot scale prototype solar photoreactor: optimization and modeling using ANN and RSM based on Box–Wilson approach. Top. Catal..

[cit55] Montgomery D. C. , Peck E. A. and Vining G. G., Introduction to Linear Regression Analysis, John Wiley & Sons, 2021

[cit56] Ali N. (2020). *et al.*, Selenide-chitosan as high-performance nanophotocatalyst for accelerated degradation of pollutants. Chem.–Asian J..

[cit57] Ahmad W. (2021). *et al.*, Photocatalytic degradation of crystal violet dye under sunlight by chitosan-encapsulated ternary metal selenide microspheres. Environ. Sci. Pollut. Res..

[cit58] Ahmad I. (2017). *et al.*, Visible light activated degradation of organic pollutants using zinc–iron selenide. J. Mol. Liq..

[cit59] Ali N. (2020). *et al.*, Regenerable chitosan-bismuth cobalt selenide hybrid microspheres for mitigation of organic pollutants in an aqueous environment. Int. J. Biol. Macromol..

[cit60] Razamin N. A. Y., Woo H. J., Winie T. (2022). Comparative study of nickel selenide, iron selenide and platinum on triiodide reduction for dye-sensitized solar cells. Opt. Mater.: X.

[cit61] Chen S. (2023). *et al.*, One-Pot Synthesis of NiSe2 with Layered Structure for Nickel-Zinc Battery. Molecules.

[cit62] Wang T. (2025). *et al.*, Synergistic role of nickel-chromium heterogeneous selenides in the efficient stabilisation of the oxygen evolution reaction. J. Alloys Compd..

[cit63] Beena V. (2021). *et al.*, Enhanced Photocatalytic and Antibacterial Activities of ZnSe Nanoparticles. J. Inorg. Organomet. Polym. Mater..

[cit64] Saied E. (2023). *et al.*, Aspergillus terreus-Mediated Selenium Nanoparticles and Their Antimicrobial and Photocatalytic Activities. Crystals.

[cit65] Al-Zaqri N. (2022). *et al.*, Green synthesis of nickel oxide nanoparticles and its photocatalytic degradation and antibacterial activity. J. Mater. Sci.: Mater. Electron..

[cit66] Smith M. C. (2017). *et al.*, Zeta potential: a case study of cationic, anionic, and neutral liposomes. Anal. Bioanal. Chem..

[cit67] Somaghian S. A. (2024). *et al.*, Biogenic zinc selenide nanoparticles fabricated using Rosmarinus officinalis leaf extract with potential biological activity. BMC Complementary Med. Ther..

[cit68] Eltaweil A. S. (2024). *et al.*, Engineering a sustainable cadmium sulfide/polyethyleneimine-functionalized biochar/chitosan composite for effective chromium adsorption: optimization, co-interfering anions, and mechanisms. RSC Adv..

[cit69] Anitha A. (2025). *et al.*, Novel biosynthesized zinc selenite photocatalysts for enhanced degradation of oxytetracycline and Rhodamine B dye with antibacterial activity. BioMetals.

[cit70] Ali N. (2020). *et al.*, Photocatalytic degradation of congo red dye from aqueous environment using cobalt ferrite nanostructures: development, characterization, and photocatalytic performance. Water, Air, Soil Pollut..

[cit71] Rahman M. U. (2021). *et al.*, Solar driven photocatalytic degradation potential of novel graphitic carbon nitride based nano zero-valent iron doped bismuth ferrite ternary composite. Opt. Mater..

[cit72] Salama A. (2018). *et al.*, Photocatalytic
degradation of organic dyes using composite nanofibers under UV irradiation. Appl. Nanosci..

[cit73] Al-Farraj E. S., Abdelrahman E. A. (2024). Efficient photocatalytic degradation of congo red dye using facilely synthesized and characterized MgAl2O4 nanoparticles. ACS Omega.

[cit74] Shaban M. (2017). *et al.*, Photocatalytic degradation and photo-Fenton oxidation of Congo red dye pollutants in water using natural chromite—response surface optimization. Appl. Water Sci..

[cit75] Ali N. (2025). *et al.*, Visible Light-Triggered Chitosan Supported Ternary Ferrite for Efficient Removal of Organic Dye Pollutants from Wastewater. Top. Catal..

[cit76] Erdemoğlu S. (2008). *et al.*, Photocatalytic degradation of Congo Red by hydrothermally synthesized nanocrystalline TiO2 and identification of degradation products by LC–MS. J. Hazard. Mater..

[cit77] Chen X. (2017). *et al.*, Preparation of ZnO Photocatalyst for the Efficient and Rapid Photocatalytic Degradation of Azo Dyes. Nanoscale Res. Lett..

[cit78] Kumar A. P. (2021). *et al.*, Photocatalytic degradation of organic dyes: Pd-γ-Al2O3 and PdO-γ-Al2O3 as potential photocatalysts. RSC Adv..

[cit79] You Q. (2025). *et al.*, Functionally graded chitosan ferrite beads for photocatalytic degradation of Eriochrome Black T and Congo red dyes. Biomass Convers. Biorefin..

[cit80] Wang Y. (2022). *et al.*, Ti3C2 MXene coupled with CdS nanoflowers as 2D/3D heterostructures for enhanced photocatalytic hydrogen production activity. Int. J. Hydrogen Energy.

[cit81] Ahmad W. (2021). *et al.*, Photocatalytic degradation of crystal violet dye under sunlight by chitosan-encapsulated ternary metal selenide microspheres. Environ. Sci. Pollut. Res..

[cit82] Altaf S. (2020). *et al.*, Synthesis and characterization of binary selenides of transition metals to investigate its photocatalytic, antimicrobial and anticancer efficacy. Appl. Nanosci..

[cit83] Butler M. A., Ginley D. S. (1978). Prediction of flatband potentials at semiconductor-electrolyte interfaces from atomic electronegativities. J. Electrochem. Soc..

[cit84] Xu Y., Schoonen M. A. (2000). The absolute energy positions of conduction and valence bands of selected semiconducting minerals. Am. Mineral..

