# Correction: Synthesis, characterization and photocatalytic evaluation of ternary metal–selenide-chitosan microspheres for the degradation of Congo red dye

**DOI:** 10.1039/d6ra90108e

**Published:** 2026-08-20

**Authors:** Nawaz Khan, Waqar Ahmad, Muhammad Khan, Mahnoor Amjad, Adnan Khan, Sabir Khan, Nisar Ali, Nauman Ali, Jiaojiao Zheng

**Affiliations:** a Institute of Chemical Sciences, University of Peshawar Khyber Pakhtunkhwa 25120 Pakistan; b Energy & Bioproducts Research Institute (EBRI), College of Engineering and Physical Sciences (EPS), Aston University Aston Triangle Birmingham B4 7ET UK j.zheng2@aston.ac.uk; c Center of Chemical, Pharmaceutical and Food Sciences, Federal University of Pelotas Pelotas 96010-900 RS Brazil; d Materials Science and Engineering (PPGCEM), Technological Development Center, Federal, University of Pelotas (UFPel) Pelotas 96010-610 RS Brazil; e Department of Basic and Applied Sciences, College of Applied and Health Sciences, A'Sharqiyah University P.O. Box 42, Ibra, P.O. 400 Sultanate of Oman

## Abstract

Correction for ‘Synthesis, characterization and photocatalytic evaluation of ternary metal–selenide-chitosan microspheres for the degradation of Congo red dye’ by Nawaz Khan *et al.*, *RSC Adv.*, 2026, https://doi.org/10.1039/d6ra04309g.

The funding information was incorrectly shown in the Acknowledgements section of the original manuscript. The corrected funding acknowledgement is as shown below.

The authors acknowledge the funding support by the Higher Education of Pakistan (NRPU Project No. 1110). Jiaojiao Zheng was funded by the Engineering and Physical Sciences Research Council grant number UKRI633.

The Royal Society of Chemistry apologises for these errors and any consequent inconvenience to authors and readers.

