# Supplementary material for: Synthesis, characterization and photocatalytic evaluation of ternary metal–selenide-chitosan microspheres for the degradation of Congo red dye
Source: RSC Adv. 2026 Jul 31;16(39):44201–16. doi: 10.1039/d6ra04309g (PMC13426363; doi:10.1039/d6ra04309g)
Supplement: Supplementary file 1 [file RA-016-D6RA04309G-s001.pdf]

Supplementary Information (SI) for RSC Advances.
This journal is © The Royal Society of Chemistry 2026

Synthesis, Characterization and Photocatalytic Evaluation of Ternary Metal-Selenide-Chitosan Microspheres for the Degradation of Congo Red Dye

Nawaz Khan [a], Waqar Ahmad [a], Muhammad Khan [b], Mahnoor Amjid [a], Adnan Khan [a,c], Sabir Khan [d], Nisar Ali [e], Nauman Ali [a], and Jiaojiao Zheng [b,*]

[a] Institute of Chemical Sciences, University of Peshawar, Khyber Pakhtunkhwa 25120, Pakistan

[b] Energy & Bioproducts Research Institute (EBRI), College of Engineering and Physical Sciences (EPS), Aston University, Aston Triangle, Birmingham, B4 7ET, United Kingdom (UK)

[c] Center of Chemical, Pharmaceutical and Food Sciences, Federal University of Pelotas, Pelotas, 96010-900, RS, Brazil

[d] Materials Science and Engineering (PPGCEM), Technological Development Center, Federal, University of Pelotas (UFPel), RS, 96010-610, Pelotas, Brazil

[e] Department of Basic and Applied Sciences, College of Applied and Health Sciences, A'Sharqiyah University, P.O. Box 42, Ibra P.O. 400, Sultanate of Oman.

* Corresponding author: Jiaojiao Zheng, j.zheng2@aston.ac.uk

1. Comparison of the photocatalytic data for various catalysts.

Table S1 highlighting its competitive degradation efficiency under sunlight conditions. These findings indicate that the developed photocatalyst exhibits promising potential for practical wastewater treatment applications.

Table S1: Relating Current Findings to Prior Research

Sl no	Photocatalyst	Methods	Irradiation Time (min)	Degradation (%)	Light Sources	References
1	ZBiSe-CM	Solvothermal	120	99.63	Solar light	[1]
2	FeNiSe-CHM	Solvothermal method	140	99	Sunlight	[2]
3	$AgInSbSe_3$	Hydrothermal route		77.8	Sunlight	[3]
4	BCSN-CM	Solvothermal process	100	85	Sunlight	[4]
5	Bi_2Se_3 and Ni doped Bi_2Se_3	Solvothermal method.	120, 90	35, 94	Visible light	[5]
6	Quaternary metal selenide	Hydrothermal process		86.9	Sunlight	[6]
7	$MgAl_2O_4$ nanoparticles	Pechini sol-gel process	80	99.27	3.71eV	[7]
8	HHCDs @TiO_2	One-pot hydrothermal process,	80	97.1	Visible light	[8]
9	$Ag_2O/TiO_2/$ g-C_3N_4	Thermal polymerization	70	85	Visible light	[9]

10	CdO, SnO_2, and CdO–SnO_2 nanocomposites	Precipitation method	80 min	92.77	UV light	[10]
11	Doped zinc oxide nanoparticles	One-step liquid impregnation	120	ZC 70, ANZ 99.7, AZ 92.5, NZ 85.8	UV light	[11]
12	Ni-Al/AC Layered Double Hydroxide	Co-precipitation method	30	91.3	UV light	[12]
13	$SrFe_{0.5}$ $Co_{0.5}O_{3-\delta}$	Citric acid method	120	97.96	UV /Visible	[13]
14	ZnO/graphitic carbon nitride		120	98.7	Sunlight.	[14]
15	Fe_3O_4/AC@ZnO composite	Activated carbon was produced from bamboo utilizing KOH as an activator	80	99.20	Visible light	[15]
16	(Ag NPs)	Biosynthesized	8,<br>54	98.9<br>97.8	Visible light,	[16]
17	TMS-CM	Co-precipitation	60	97	Sunlight	This Study

2. Photocatalytic mechanism

The helpful processes shown in the Introduction section are based on the in-situ production of non-selective radicals with a strong oxidation power able to mineralize the organic pollutants [17-19]. Photocatalytic processes, which are newly emerging AOPs, have been extensively used for the degradation of different dye-stuffs in wastewater [20]. Photocatalysis has emerged as a desirable technology for the treatment of wastewater and is considered an efficient, eco-friendly, and cost-effective method to resolve problems of environmental pollution [19, 21, 22].

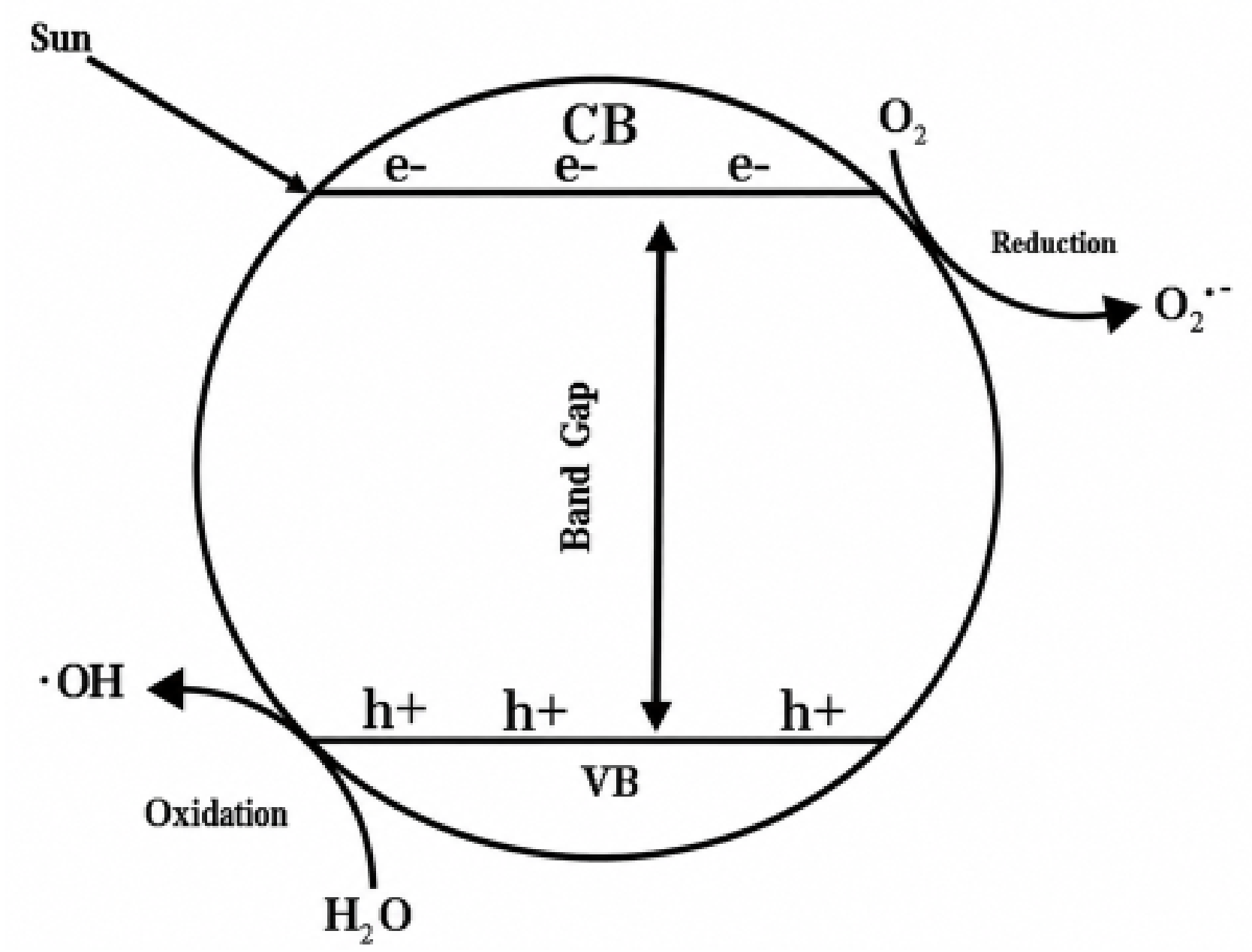

Figure S1. Photocatalytic Electron-Hole Pair Generation under Light Irradiation [23].

As shown in Fig. S1, in the photocatalysis process, a light source activates the catalyst that creates electron **(e^-)** and hole (h^+) pairs. The (h^+) reacts with water molecules to produce hydroxyl radicals ($^{\bullet}OH$). while **(e^-)** reacts with surface oxygen to form superoxide radicals ($O_2^{\bullet-}$). These radicals and holes with Redox potential degrade of the concerned contaminants and form water molecules, carbon dioxide and transformed products [24, 25]. Numerous photocatalysts have been employed to degrade different types of pollutants [26].

The photocatalytic degradation mechanism involves the excitation of electrons from the valence band to the conduction band upon light irradiation, resulting in the generation of electron-hole pairs. The photogenerated holes react with water molecules to produce hydroxyl radicals (•OH), while the electrons reduce dissolved oxygen to form superoxide radicals. These reactive oxygen species subsequently undergo a series of reactions, leading to the formation of highly oxidative intermediates capable of mineralizing dye molecules into harmless end products such as carbon dioxide and water. The overall degradation pathway is illustrated in (Equations S1-7) and schematically represented in Figure S2 [73].

$$Catalyst + hv \rightarrow Catalyst\ (e_{cb}^{-} + h_{vb}^{+})$$ *Equation S1*

$$H_2O + h_{vb}^{+} \rightarrow OH^{\cdot} + H^{+}$$ *Equation S2*

$$O_2 + e_{cb}^{-} \rightarrow O_2^{-\cdot}$$ *Equation S3*

$$O_2^{-\cdot} + H^{+} \rightleftarrows HOO^{\cdot}$$ *Equation S4*

$$2HOO^{\cdot} \rightarrow H_2O_2 + O_2$$ *Equation S5*

$$H_2O_2 \rightarrow 2OH^{\cdot}$$ *Equation S6*

$$Dye + OH^{\cdot} \rightarrow CO_2 + H_2O(dye\ intermediates)$$ *Equation S7*

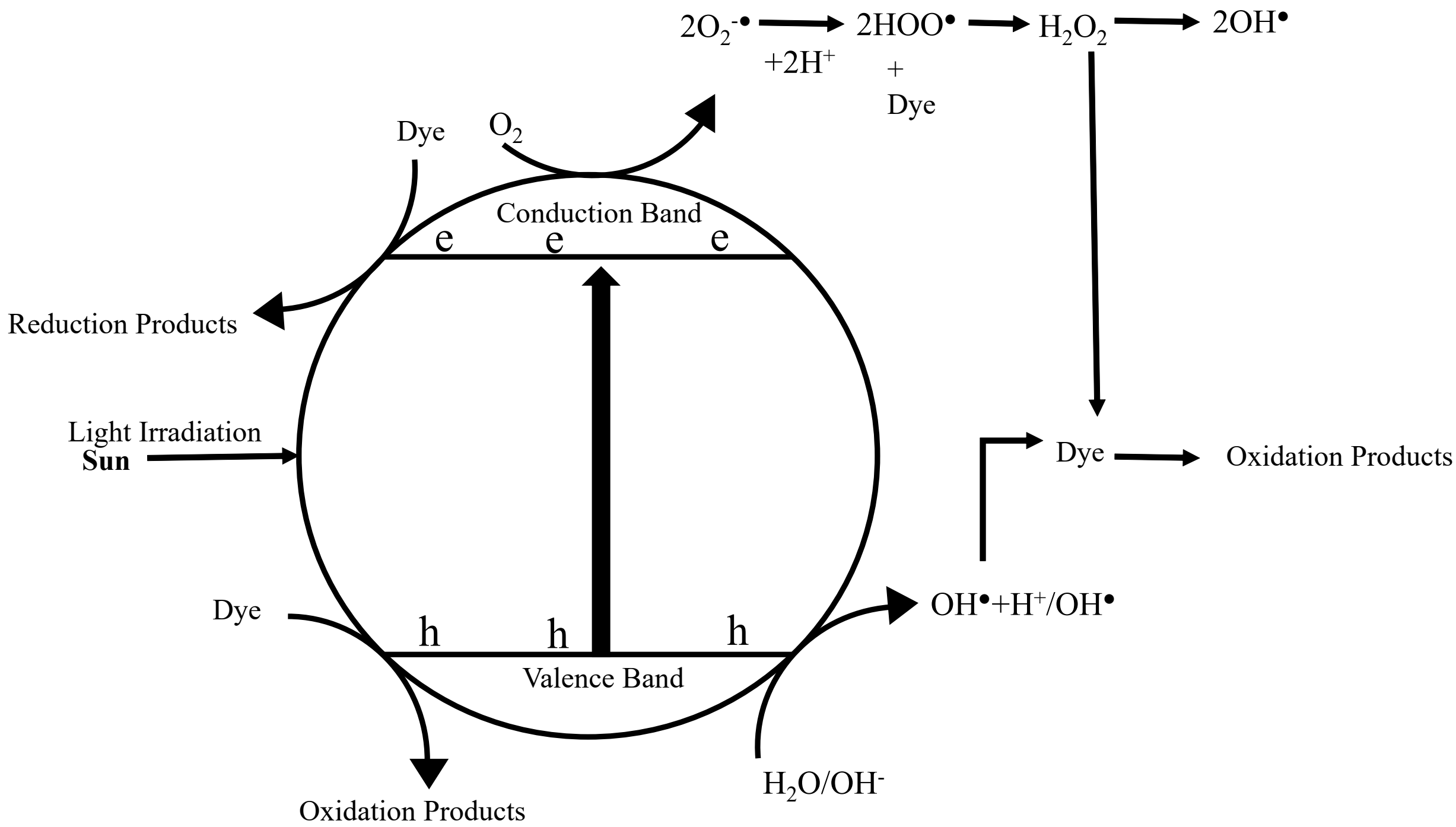

Figure S2. Photocatalytic degradation mechanism schematics [27].

Lately, there has been considerable advancement in the field of photocatalytic degradation by the production of selenide compounds. These selenide compounds have demonstrated significant promise for effective organic pollutant photocatalytic decontamination. A key advantage of metal selenide nanoparticles lies in their intrinsically narrow bandgap, typically below 3 eV, which enables efficient absorption of visible light within the solar spectrum. This electronic structure facilitates enhanced photoexcitation under visible irradiation, thereby improving photocatalytic performance compared to wide bandgap materials. Furthermore, their nanoscale dimensions provide a high surface-to-volume ratio, resulting in an increased density of accessible active sites that promote interfacial redox reactions. Collectively, these attributes render metal selenide nanomaterials highly effective for visible-light-driven photocatalytic applications [1, 28].

To further improve the photocatalytic performance of ternary metal selenides, the incorporation of a suitable biopolymeric support has emerged as an effective strategy. Biopolymer nanoparticle composites have gained considerable attention due to their

ability to synergistically enhance the intrinsic properties of the embedded nanomaterials, including improved dispersion, stability, and accessibility of active sites. Naturally derived polymers such as cellulose and chitosan have been widely explored in this context owing to their biocompatibility, abundance, and functional versatility. Among these, chitosan has proven particularly advantageous as a composite matrix for metal selenides and metal oxides, primarily due to the presence of abundant amino and hydroxyl functional groups that facilitate strong interactions with nanoparticle surfaces, thereby promoting uniform distribution and enhanced photocatalytic efficiency [18, 29, 30]. Owing to its excellent biocompatibility, biodegradability, and non-toxic nature, chitosan has been extensively employed as a stabilizing, capping, and supporting matrix for metal selenide nanoparticles [31, 32]. The incorporation of nanoparticles within the chitosan framework not only enhances their structural stability and dispersion but also facilitates convenient separation and recovery from reaction systems, thereby improving the overall practicality and reusability of the photocatalytic material [4].

Reference

1. Khan, S., et al., *Degradation of Congo red dye using ternary metal selenide-chitosan microspheres as robust and reusable catalysts.* Environmental Technology & Innovation, 2021. **22**: p. 101402.
2. Yang, Y., et al., *Chitosan-capped ternary metal selenide nanocatalysts for efficient degradation of Congo red dye in sunlight irradiation.* International Journal of Biological Macromolecules, 2021. **167**: p. 169-181.
3. Yasmin, N., et al., *Synthesis and characterization of silver-indium and antimony selenide: role in photocatalytic degradation of dyes.* Heliyon, 2022. **8**(10).
4. Ali, N., et al., *Regenerable chitosan-bismuth cobalt selenide hybrid microspheres for mitigation of organic pollutants in an aqueous environment.* International Journal of Biological Macromolecules, 2020. **161**: p. 1305-1317.
5. Kulsi, C., et al., *Enhanced photo catalytic performance of nickel doped bismuth selenide under visible light irradiation.* Materials Research Express, 2017. **4**(3): p. 035902.
6. Yasmin, N., et al., *Synthesis, characterization, and photocatalytic activity of quaternary selenide composite using natural sunlight.* Optik, 2022. **251**: p. 168460.
7. Al-Farraj, E.S. and E.A. Abdelrahman, *Efficient Photocatalytic Degradation of Congo Red Dye Using Facilely Synthesized and Characterized MgAl2O4 Nanoparticles.* ACS Omega, 2024. **9**(4): p. 4870-4880.
8. Hamasalih, G.H., S.J. Mohammed, and S.B. Aziz, *Sustainable TiO2 photocatalysts modified with hollyhock-derived carbon dots and natural dye for enhanced visible-light degradation of Congo red: a comparative study.* Materials Advances, 2026. **7**(2): p. 913-932.
9. Prabakaran, E. and K. Pillay, *Silver and Titanium Oxides Coated on g-C3N4 Nanocomposite for Photocatalytic Degradation of Mixture of Brilliant Green-Congo Red Dyes and Ciprofloxacin Antibiotic Under Visible Light Irradiation.* Water Conservation Science and Engineering, 2025. **10**(1): p. 9.
10. Nawin, L., N. Khan, and C. Thakur, *Enhanced photocatalytic degradation of Congo Red Dye by CdO, SnO2, and CdO–SnO2 nanocomposite under UV light.* Indian Chemical Engineer, 2025: p. 1-15.
11. Nwaiwu, B.C., E.E. Oguzie, and C.C. Ejiogu, *Photocatalytic degradation of Congo red using doped zinc oxide nanoparticles.* EQA-International Journal of Environmental Quality, 2024. **60**: p. 18-26.
12. Baba, C. and A.S. Muhammad, *Photocatalytic Degradation of Congo Red Using Ni-Al/AC Layered Double Hydroxide under UV Light Irradiation.* Applied Journal of Environmental Engineering Science, 2023. **9**(3): p. & al./Appl. J. Envir. Eng. Sci. 9 N° 3 (2023) 163-176.
13. Li, C., et al., *The Photocatalytic Activity of Degrading Congo Red Using SrFe0. 5Co0. 5O3-δ.* Advanced Materials Research, 2014. **968**: p. 58-62.

14. Priyadarshini, U., Y.N. Priya, and R. Neelancherry, *Solar photocatalytic degradation of congo red from aqueous phase using modified ZnO/graphite carbon nitride nanocomposites.* Journal of Water Process Engineering, 2026. **83**: p. 109669.
15. Riyanti, F., et al., *ZnO doped Fe3O4/Activated carbon Composite for High Efficient Photocatalytic Degradation of Congo red Dye.* Egyptian Journal of Chemistry, 2025. **68**(12): p. 213-224.
16. Aslan, T.N., *Visible Light Driven Photocatalytic Degradation of Congo Red Dye Using Biogenic Silver Nanoparticles.* Cumhuriyet Science Journal, 2025. **46**(3): p. 532-539.
17. Fdez-Sanromán, A., et al., *Metal–organic frameworks as powerful heterogeneous catalysts in advanced oxidation processes for wastewater treatment.* Applied Sciences, 2022. **12**(16): p. 8240.
18. Nawaz, A., et al., *Fabrication and characterization of new ternary ferrites-chitosan nanocomposite for solar-light driven photocatalytic degradation of a model textile dye.* Environmental Technology & Innovation, 2020. **20**: p. 101079.
19. Alhokbany, N.S., et al., *Fabrication of Z-scheme photocatalysts g-C3N4/Ag3PO4/chitosan for the photocatalytic degradation of ciprofloxacin.* International journal of biological macromolecules, 2020. **164**: p. 3864-3872.
20. Tanaka, K., K. Padermpole, and T. Hisanaga, *Photocatalytic degradation of commercial azo dyes.* Water Research, 2000. **34**(1): p. 327-333.
21. Ali, N., et al., *Photocatalytic Degradation of Congo Red Dye from Aqueous Environment Using Cobalt Ferrite Nanostructures: Development, Characterization, and Photocatalytic Performance.* Water, Air, & Soil Pollution, 2020. **231**(2): p. 50.
22. Mahanta, S.K., et al., *Direct fabrication of metal-free graphene nanohairs/polyimide heterojunction for the highly efficient photocatalytic degradation of industrial dyes.* Diamond and Related Materials, 2023. **137**: p. 110096.
23. Chakravorty, A. and S. Roy, *A review of photocatalysis, basic principles, processes, and materials.* Sustainable Chemistry for the Environment, 2024. **8**: p. 100155.
24. Gupta, B. and A.K. Gupta, *Photocatalytic performance of 3D engineered chitosan hydrogels embedded with sulfur-doped C3N4/ZnO nanoparticles for Ciprofloxacin removal: Degradation and mechanistic pathways.* International Journal of Biological Macromolecules, 2022. **198**: p. 87-100.
25. Lotfi, S., et al., *Recent progress on the synthesis, morphology and photocatalytic dye degradation of BiVO4 photocatalysts: A review.* Catalysis Reviews, 2024. **66**(1): p. 214-258.
26. Ali, N., et al., *Selenide-chitosan as high-performance nanophotocatalyst for accelerated degradation of pollutants.* Chemistry–An Asian Journal, 2020. **15**(17): p. 2660-2673.

27. Ajmal, A., et al., *Principles and mechanisms of photocatalytic dye degradation on TiO 2 based photocatalysts: a comparative overview.* Rsc Advances, 2014. **4**(70): p. 37003-37026.
28. Azam, J., et al., *Solar Light Assisted Metal Selenide-Chitosan Nanoparticles from Structure Tailoring to Photocatalytic Mineralization of Selected Industrial Dyes.* Water, Air, & Soil Pollution, 2024. **235**(6): p. 386.
29. Ahmad, W., et al., *Photocatalytic degradation of crystal violet dye under sunlight by chitosan-encapsulated ternary metal selenide microspheres.* Environmental Science and Pollution Research, 2021. **28**(7): p. 8074-8087.
30. Nawaz, A., et al., *Synthesis of ternary-based visible light nano-photocatalyst for decontamination of organic dyes-loaded wastewater.* Chemosphere, 2022. **289**: p. 133121.
31. Khan, M., et al., *Development and characterization of regenerable chitosan-coated nickel selenide nano-photocatalytic system for decontamination of toxic azo dyes.* International journal of biological macromolecules, 2021. **182**: p. 866-878.
32. Aziz, A., et al., *Chitosan-zinc sulfide nanoparticles, characterization and their photocatalytic degradation efficiency for azo dyes.* International journal of biological macromolecules, 2020. **153**: p. 502-512.
